# Supplementary material for: Health and social behaviour through pandemic phases in Switzerland: Regional time-trends of the COVID-19 Social Monitor panel study
Source: PLoS One. 2021 Aug 25;16(8):e0256253. doi: 10.1371/journal.pone.0256253 (PMC8386858; doi:10.1371/journal.pone.0256253)
Supplement: S1 Table — (DOCX) [file pone.0256253.s001.docx]

**S1 Table.** The domain, the source and the original question from the survey questionnaire.

| **Domain** | **Source** | **Original survey question (only in German)** |
| --- | --- | --- |
| General health | Swiss Health Survey | Wie ist Ihr Gesundheitszustand im Allgemeinen? |
| Quality of life | Swiss Health Survey | Wie schätzen Sie Ihre Lebensqualität im Allgemeinen im Moment ein? |
| Mental health: Depressive mood | - | Wie haben Sie sich in den letzten 7 Tagen gefühlt? 1) So niedergeschlagen oder verstimmt, dass Sie nichts hat aufmuntern können 2) Entmutigt und deprimiert |
| Mental health: Lack of energy | - | Wie haben Sie sich in den letzten 7 Tagen gefühlt? Voller Energie |
| Mental health: Fear of losing job | Swiss Health Survey | Haben Sie Angst, Ihren heutigen Arbeitsplatz zu verlieren? |
| Social interaction: Loneliness | Swiss Health Survey | Wie häufig kommt es momentan vor, dass Sie sich einsam fühlen? |
| Social interaction: Isolation | - | Wie oft fühlen Sie sich von anderen isoliert? (Population 65 Jahre oder älter) |
| Physical activity | doi:10.1016/j.ypmed.2014.01.025 | An wie vielen Tagen der letzten Woche waren Sie insgesamt 30 Minuten oder länger körperlich aktiv, so dass Sie zumindest etwas stärker atmen mussten? |
| Health care use | partly Swiss Health Survey | Haben Sie in den letzten 14 Tagen eine medizinische Behandlung erhalten? |
| Health care non-use | partly Swiss Health Survey | Konnten Sie in den letzten 14 Tagen eine geplante oder nötige medizinische Behandlung nicht in Anspruch nehmen wegen der Corona-Krise? |
| Health care use due to COVID-19 | - | Haben Sie in den letzen 7 Tagen einen Arzt oder ein Spital kontaktiert wegen einem Gesundheitsproblem von Ihnen, das mit dem Corona-Virus zu tun hatte? |
| Mitigation measures | - | Inwiefern haben Sie folgende Massnahmen ergriffen, um sich und Andere vor dem Corona-Virus zu schützen? 2 Meter Abstand zu anderen Personen eingehalten, Gesichtsmaske getragen, Private Verabredungen/Besuche unterlassen, Öffentlichen Verkehr gemieden |
