# Supplementary material for: Health and social behaviour through pandemic phases in Switzerland: Regional time-trends of the COVID-19 Social Monitor panel study
Source: PLoS One. 2021 Aug 25;16(8):e0256253. doi: 10.1371/journal.pone.0256253 (PMC8386858; doi:10.1371/journal.pone.0256253)
Supplement: S2 Table — (DOCX) [file pone.0256253.s002.docx]

**S3 Table.** Description of missing values.

|  | Subpopulation | Missing values | Denominator | Percentage | Replaced by (median) |
| --- | --- | --- | --- | --- | --- |
| Baseline characteristics |  |  |  |  |  |
| Citizenship |  | 5 | 3381 | 0.1% | Swiss |
| Education |  | 15 | 3381 | 0.4% | Secondary |
| Study outcomes |  |  |  |  |  |
| General health status |  | 4 | 29771 | 0.01% | Very good/good/fair health status |
| Self-assessed quality of life |  | 2 | 29771 | 0.01% | Very good/good/fair quality of life |
| Depressive mood |  | 43 | 29771 | 0.14% | No depressive mood |
| Always lack of energy |  | 28 | 29771 | 0.09% | No lack of energy |
| Fear of losing employment | Working population | 3 | 20823 | 0.01% | No fear |
| Feelings of loneliness |  | 6 | 29771 | 0.02% | No feelings of loneliness |
| Feelings of isolation | >=65 years | 5 | 12244 | 0.04% | No feelings of isolation |
| Physical activity |  | 19 | 29771 | 0.06% | Phyiscal active |
| Health care use |  | 6 | 29771 | 0.02% | No use |
| Health care non-use |  | 0 | 29771 | - | - |
| COVID-19 related health care use |  | 0 | 29771 | - | - |
| Always adherence to physical distance when meeting persons | Wave 2 onwards | 14 | 27745 | 0.05% | Not always |
| Always the wearing of face masks | Wave 2 onwards | 17 | 27745 | 0.06% | Not always |
| Always avoidance of private appointments | Wave 2 onwards | 20 | 27745 | 0.07% | Not always |
| Always non-use of public transport | Wave 2 onwards | 20 | 27745 | 0.07% | Always |
