# Supplementary material for: Health and social behaviour through pandemic phases in Switzerland: Regional time-trends of the COVID-19 Social Monitor panel study
Source: PLoS One. 2021 Aug 25;16(8):e0256253. doi: 10.1371/journal.pone.0256253 (PMC8386858; doi:10.1371/journal.pone.0256253)
Supplement: S3 Table — (DOCX) [file pone.0256253.s003.docx]

**S4 Table.** Underlying results for Figures 3-5.

| **Study outcome** | **Language region** | **Period** | **No. of answers** | **Positive answers** | **Weighted proportion** | **Lower 95% CI** | **Upper 95% CI** | **Crude proportion** |
| --- | --- | --- | --- | --- | --- | --- | --- | --- |
| Poor health status | German/Romansh | March 16, 2020 to May 10, 2020 | 4383 | 44 | 0.009 | 0.006 | 0.015 | 0.010 |
| Poor health status | French | March 16, 2020 to May 10, 2020 | 1460 | 13 | 0.008 | 0.004 | 0.015 | 0.009 |
| Poor health status | Italian | March 16, 2020 to May 10, 2020 | 989 | 10 | 0.012 | 0.004 | 0.035 | 0.010 |
| Poor health status | German/Romansh | May 11, 2020 to July 5, 2020 | 3118 | 29 | 0.009 | 0.005 | 0.013 | 0.009 |
| Poor health status | French | May 11, 2020 to July 5, 2020 | 1018 | 10 | 0.009 | 0.003 | 0.021 | 0.010 |
| Poor health status | Italian | May 11, 2020 to July 5, 2020 | 675 | 9 | 0.015 | 0.006 | 0.038 | 0.013 |
| Poor health status | German/Romansh | July 6, 2020 to October 18, 2020 | 2973 | 45 | 0.015 | 0.010 | 0.022 | 0.015 |
| Poor health status | French | July 6, 2020 to October 18, 2020 | 949 | 12 | 0.012 | 0.005 | 0.026 | 0.013 |
| Poor health status | Italian | July 6, 2020 to October 18, 2020 | 629 | 12 | 0.021 | 0.009 | 0.049 | 0.019 |
| Poor health status | German/Romansh | October 19, 2020 to January 17, 2021 | 4433 | 77 | 0.017 | 0.012 | 0.022 | 0.017 |
| Poor health status | French | October 19, 2020 to January 17, 2021 | 1421 | 30 | 0.020 | 0.012 | 0.032 | 0.021 |
| Poor health status | Italian | October 19, 2020 to January 17, 2021 | 1004 | 27 | 0.029 | 0.017 | 0.047 | 0.027 |
| Poor health status | German/Romansh | January 18, 2021 onwards | 4400 | 87 | 0.020 | 0.015 | 0.026 | 0.020 |
| Poor health status | French | January 18, 2021 onwards | 1341 | 28 | 0.019 | 0.011 | 0.033 | 0.021 |
| Poor health status | Italian | January 18, 2021 onwards | 978 | 20 | 0.020 | 0.011 | 0.038 | 0.020 |
| Poor quality of life | German/Romansh | March 16, 2020 to May 10, 2020 | 4383 | 103 | 0.022 | 0.017 | 0.029 | 0.023 |
| Poor quality of life | French | March 16, 2020 to May 10, 2020 | 1460 | 19 | 0.013 | 0.007 | 0.024 | 0.013 |
| Poor quality of life | Italian | March 16, 2020 to May 10, 2020 | 989 | 9 | 0.010 | 0.004 | 0.024 | 0.009 |
| Poor quality of life | German/Romansh | May 11, 2020 to July 5, 2020 | 3118 | 61 | 0.019 | 0.013 | 0.027 | 0.020 |
| Poor quality of life | French | May 11, 2020 to July 5, 2020 | 1018 | 14 | 0.013 | 0.007 | 0.025 | 0.014 |
| Poor quality of life | Italian | May 11, 2020 to July 5, 2020 | 675 | 3 | 0.004 | 0.001 | 0.019 | 0.004 |
| Poor quality of life | German/Romansh | July 6, 2020 to October 18, 2020 | 2973 | 58 | 0.019 | 0.013 | 0.027 | 0.020 |
| Poor quality of life | French | July 6, 2020 to October 18, 2020 | 949 | 22 | 0.022 | 0.012 | 0.038 | 0.023 |
| Poor quality of life | Italian | July 6, 2020 to October 18, 2020 | 629 | 2 | 0.003 | 0.001 | 0.014 | 0.003 |
| Poor quality of life | German/Romansh | October 19, 2020 to January 17, 2021 | 4433 | 185 | 0.040 | 0.033 | 0.048 | 0.042 |
| Poor quality of life | French | October 19, 2020 to January 17, 2021 | 1421 | 48 | 0.033 | 0.022 | 0.049 | 0.034 |
| Poor quality of life | Italian | October 19, 2020 to January 17, 2021 | 1004 | 22 | 0.020 | 0.012 | 0.033 | 0.022 |
| Poor quality of life | German/Romansh | January 18, 2021 onwards | 4400 | 141 | 0.032 | 0.026 | 0.039 | 0.032 |
| Poor quality of life | French | January 18, 2021 onwards | 1341 | 38 | 0.028 | 0.018 | 0.044 | 0.028 |
| Poor quality of life | Italian | January 18, 2021 onwards | 978 | 15 | 0.016 | 0.008 | 0.032 | 0.015 |
| Depressive mood | German/Romansh | March 16, 2020 to May 10, 2020 | 4383 | 222 | 0.047 | 0.039 | 0.056 | 0.051 |
| Depressive mood | French | March 16, 2020 to May 10, 2020 | 1460 | 80 | 0.055 | 0.041 | 0.072 | 0.055 |
| Depressive mood | Italian | March 16, 2020 to May 10, 2020 | 989 | 81 | 0.077 | 0.055 | 0.106 | 0.082 |
| Depressive mood | German/Romansh | May 11, 2020 to July 5, 2020 | 3118 | 153 | 0.047 | 0.038 | 0.057 | 0.049 |
| Depressive mood | French | May 11, 2020 to July 5, 2020 | 1018 | 62 | 0.061 | 0.044 | 0.084 | 0.061 |
| Depressive mood | Italian | May 11, 2020 to July 5, 2020 | 675 | 47 | 0.066 | 0.045 | 0.095 | 0.070 |
| Depressive mood | German/Romansh | July 6, 2020 to October 18, 2020 | 2973 | 127 | 0.041 | 0.033 | 0.052 | 0.043 |
| Depressive mood | French | July 6, 2020 to October 18, 2020 | 949 | 48 | 0.050 | 0.034 | 0.071 | 0.051 |
| Depressive mood | Italian | July 6, 2020 to October 18, 2020 | 629 | 33 | 0.052 | 0.033 | 0.081 | 0.052 |
| Depressive mood | German/Romansh | October 19, 2020 to January 17, 2021 | 4433 | 301 | 0.064 | 0.056 | 0.074 | 0.068 |
| Depressive mood | French | October 19, 2020 to January 17, 2021 | 1421 | 114 | 0.080 | 0.062 | 0.102 | 0.080 |
| Depressive mood | Italian | October 19, 2020 to January 17, 2021 | 1004 | 107 | 0.102 | 0.080 | 0.131 | 0.107 |
| Depressive mood | German/Romansh | January 18, 2021 onwards | 4400 | 253 | 0.057 | 0.049 | 0.067 | 0.058 |
| Depressive mood | French | January 18, 2021 onwards | 1341 | 104 | 0.080 | 0.062 | 0.104 | 0.078 |
| Depressive mood | Italian | January 18, 2021 onwards | 978 | 98 | 0.101 | 0.077 | 0.131 | 0.100 |
| Lack of energy | German/Romansh | March 16, 2020 to May 10, 2020 | 4383 | 597 | 0.133 | 0.119 | 0.149 | 0.136 |
| Lack of energy | French | March 16, 2020 to May 10, 2020 | 1460 | 231 | 0.155 | 0.129 | 0.185 | 0.158 |
| Lack of energy | Italian | March 16, 2020 to May 10, 2020 | 989 | 146 | 0.144 | 0.114 | 0.179 | 0.148 |
| Lack of energy | German/Romansh | May 11, 2020 to July 5, 2020 | 3118 | 370 | 0.116 | 0.102 | 0.133 | 0.119 |
| Lack of energy | French | May 11, 2020 to July 5, 2020 | 1018 | 151 | 0.145 | 0.118 | 0.178 | 0.148 |
| Lack of energy | Italian | May 11, 2020 to July 5, 2020 | 675 | 93 | 0.131 | 0.101 | 0.167 | 0.138 |
| Lack of energy | German/Romansh | July 6, 2020 to October 18, 2020 | 2973 | 340 | 0.114 | 0.099 | 0.130 | 0.114 |
| Lack of energy | French | July 6, 2020 to October 18, 2020 | 949 | 124 | 0.127 | 0.100 | 0.160 | 0.131 |
| Lack of energy | Italian | July 6, 2020 to October 18, 2020 | 629 | 79 | 0.122 | 0.093 | 0.160 | 0.126 |
| Lack of energy | German/Romansh | October 19, 2020 to January 17, 2021 | 4433 | 765 | 0.169 | 0.154 | 0.184 | 0.173 |
| Lack of energy | French | October 19, 2020 to January 17, 2021 | 1421 | 290 | 0.201 | 0.175 | 0.230 | 0.204 |
| Lack of energy | Italian | October 19, 2020 to January 17, 2021 | 1004 | 196 | 0.189 | 0.160 | 0.223 | 0.195 |
| Lack of energy | German/Romansh | January 18, 2021 onwards | 4400 | 713 | 0.161 | 0.146 | 0.176 | 0.162 |
| Lack of energy | French | January 18, 2021 onwards | 1341 | 246 | 0.183 | 0.156 | 0.214 | 0.183 |
| Lack of energy | Italian | January 18, 2021 onwards | 978 | 182 | 0.189 | 0.156 | 0.227 | 0.186 |
| Fear of loosing employment | German/Romansh | March 16, 2020 to May 10, 2020 | 3178 | 269 | 0.083 | 0.068 | 0.100 | 0.085 |
| Fear of loosing employment | French | March 16, 2020 to May 10, 2020 | 998 | 109 | 0.108 | 0.079 | 0.146 | 0.109 |
| Fear of loosing employment | Italian | March 16, 2020 to May 10, 2020 | 629 | 76 | 0.121 | 0.084 | 0.172 | 0.121 |
| Fear of loosing employment | German/Romansh | May 11, 2020 to July 5, 2020 | 2238 | 154 | 0.067 | 0.053 | 0.085 | 0.069 |
| Fear of loosing employment | French | May 11, 2020 to July 5, 2020 | 700 | 71 | 0.103 | 0.074 | 0.142 | 0.101 |
| Fear of loosing employment | Italian | May 11, 2020 to July 5, 2020 | 418 | 47 | 0.113 | 0.075 | 0.166 | 0.112 |
| Fear of loosing employment | German/Romansh | July 6, 2020 to October 18, 2020 | 2155 | 151 | 0.069 | 0.055 | 0.087 | 0.070 |
| Fear of loosing employment | French | July 6, 2020 to October 18, 2020 | 653 | 64 | 0.101 | 0.071 | 0.142 | 0.098 |
| Fear of loosing employment | Italian | July 6, 2020 to October 18, 2020 | 397 | 45 | 0.118 | 0.075 | 0.181 | 0.113 |
| Fear of loosing employment | German/Romansh | October 19, 2020 to January 17, 2021 | 3192 | 249 | 0.078 | 0.066 | 0.092 | 0.078 |
| Fear of loosing employment | French | October 19, 2020 to January 17, 2021 | 962 | 82 | 0.085 | 0.063 | 0.115 | 0.085 |
| Fear of loosing employment | Italian | October 19, 2020 to January 17, 2021 | 671 | 89 | 0.135 | 0.101 | 0.178 | 0.133 |
| Fear of loosing employment | German/Romansh | January 18, 2021 onwards | 3122 | 197 | 0.064 | 0.052 | 0.078 | 0.063 |
| Fear of loosing employment | French | January 18, 2021 onwards | 886 | 56 | 0.061 | 0.042 | 0.088 | 0.063 |
| Fear of loosing employment | Italian | January 18, 2021 onwards | 624 | 81 | 0.133 | 0.097 | 0.179 | 0.130 |
| Feelings of loneliness | German/Romansh | March 16, 2020 to May 10, 2020 | 4383 | 67 | 0.014 | 0.010 | 0.020 | 0.015 |
| Feelings of loneliness | French | March 16, 2020 to May 10, 2020 | 1460 | 54 | 0.037 | 0.024 | 0.056 | 0.037 |
| Feelings of loneliness | Italian | March 16, 2020 to May 10, 2020 | 989 | 19 | 0.018 | 0.010 | 0.032 | 0.019 |
| Feelings of loneliness | German/Romansh | May 11, 2020 to July 5, 2020 | 3118 | 36 | 0.011 | 0.007 | 0.017 | 0.012 |
| Feelings of loneliness | French | May 11, 2020 to July 5, 2020 | 1018 | 27 | 0.029 | 0.016 | 0.052 | 0.027 |
| Feelings of loneliness | Italian | May 11, 2020 to July 5, 2020 | 675 | 12 | 0.016 | 0.007 | 0.036 | 0.018 |
| Feelings of loneliness | German/Romansh | July 6, 2020 to October 18, 2020 | 2973 | 27 | 0.009 | 0.005 | 0.016 | 0.009 |
| Feelings of loneliness | French | July 6, 2020 to October 18, 2020 | 949 | 24 | 0.027 | 0.016 | 0.046 | 0.025 |
| Feelings of loneliness | Italian | July 6, 2020 to October 18, 2020 | 629 | 7 | 0.010 | 0.004 | 0.026 | 0.011 |
| Feelings of loneliness | German/Romansh | October 19, 2020 to January 17, 2021 | 4433 | 72 | 0.015 | 0.011 | 0.021 | 0.016 |
| Feelings of loneliness | French | October 19, 2020 to January 17, 2021 | 1421 | 49 | 0.038 | 0.025 | 0.056 | 0.034 |
| Feelings of loneliness | Italian | October 19, 2020 to January 17, 2021 | 1004 | 27 | 0.024 | 0.014 | 0.042 | 0.027 |
| Feelings of loneliness | German/Romansh | January 18, 2021 onwards | 4400 | 70 | 0.016 | 0.011 | 0.022 | 0.016 |
| Feelings of loneliness | French | January 18, 2021 onwards | 1341 | 50 | 0.041 | 0.027 | 0.060 | 0.037 |
| Feelings of loneliness | Italian | January 18, 2021 onwards | 978 | 30 | 0.031 | 0.019 | 0.052 | 0.031 |
| Population 65 years or older: Feelings of social isolation | German/Romansh | March 16, 2020 to May 10, 2020 | 487 | 26 | 0.053 | 0.035 | 0.081 | 0.053 |
| Population 65 years or older: Feelings of social isolation | French | March 16, 2020 to May 10, 2020 | 152 | 21 | 0.147 | 0.087 | 0.238 | 0.138 |
| Population 65 years or older: Feelings of social isolation | Italian | March 16, 2020 to May 10, 2020 | 97 | 8 | 0.085 | 0.036 | 0.187 | 0.082 |
| Population 65 years or older: Feelings of social isolation | German/Romansh | May 11, 2020 to July 5, 2020 | 513 | 20 | 0.042 | 0.023 | 0.077 | 0.039 |
| Population 65 years or older: Feelings of social isolation | French | May 11, 2020 to July 5, 2020 | 157 | 17 | 0.118 | 0.059 | 0.223 | 0.108 |
| Population 65 years or older: Feelings of social isolation | Italian | May 11, 2020 to July 5, 2020 | 108 | 1 | 0.010 | 0.001 | 0.067 | 0.009 |
| Population 65 years or older: Feelings of social isolation | German/Romansh | July 6, 2020 to October 18, 2020 | 503 | 9 | 0.020 | 0.008 | 0.045 | 0.018 |
| Population 65 years or older: Feelings of social isolation | French | July 6, 2020 to October 18, 2020 | 155 | 10 | 0.072 | 0.028 | 0.172 | 0.065 |
| Population 65 years or older: Feelings of social isolation | Italian | July 6, 2020 to October 18, 2020 | 99 | 2 | 0.023 | 0.003 | 0.142 | 0.020 |
| Population 65 years or older: Feelings of social isolation | German/Romansh | October 19, 2020 to January 17, 2021 | 764 | 44 | 0.054 | 0.037 | 0.080 | 0.058 |
| Population 65 years or older: Feelings of social isolation | French | October 19, 2020 to January 17, 2021 | 236 | 21 | 0.094 | 0.052 | 0.165 | 0.089 |
| Population 65 years or older: Feelings of social isolation | Italian | October 19, 2020 to January 17, 2021 | 162 | 11 | 0.063 | 0.032 | 0.119 | 0.068 |
| Population 65 years or older: Feelings of social isolation | German/Romansh | January 18, 2021 onwards | 800 | 38 | 0.050 | 0.031 | 0.079 | 0.048 |
| Population 65 years or older: Feelings of social isolation | French | January 18, 2021 onwards | 253 | 17 | 0.072 | 0.037 | 0.136 | 0.067 |
| Population 65 years or older: Feelings of social isolation | Italian | January 18, 2021 onwards | 175 | 15 | 0.083 | 0.040 | 0.165 | 0.086 |
| No physical activity | German/Romansh | March 16, 2020 to May 10, 2020 | 4383 | 571 | 0.126 | 0.112 | 0.142 | 0.130 |
| No physical activity | French | March 16, 2020 to May 10, 2020 | 1460 | 326 | 0.219 | 0.186 | 0.256 | 0.223 |
| No physical activity | Italian | March 16, 2020 to May 10, 2020 | 989 | 206 | 0.197 | 0.159 | 0.242 | 0.208 |
| No physical activity | German/Romansh | May 11, 2020 to July 5, 2020 | 3118 | 285 | 0.089 | 0.076 | 0.103 | 0.091 |
| No physical activity | French | May 11, 2020 to July 5, 2020 | 1018 | 194 | 0.191 | 0.159 | 0.227 | 0.191 |
| No physical activity | Italian | May 11, 2020 to July 5, 2020 | 675 | 127 | 0.183 | 0.144 | 0.229 | 0.188 |
| No physical activity | German/Romansh | July 6, 2020 to October 18, 2020 | 2973 | 285 | 0.095 | 0.082 | 0.111 | 0.096 |
| No physical activity | French | July 6, 2020 to October 18, 2020 | 949 | 179 | 0.189 | 0.157 | 0.227 | 0.189 |
| No physical activity | Italian | July 6, 2020 to October 18, 2020 | 629 | 120 | 0.190 | 0.151 | 0.235 | 0.191 |
| No physical activity | German/Romansh | October 19, 2020 to January 17, 2021 | 4433 | 646 | 0.138 | 0.125 | 0.152 | 0.146 |
| No physical activity | French | October 19, 2020 to January 17, 2021 | 1421 | 371 | 0.257 | 0.228 | 0.289 | 0.261 |
| No physical activity | Italian | October 19, 2020 to January 17, 2021 | 1004 | 270 | 0.262 | 0.228 | 0.300 | 0.269 |
| No physical activity | German/Romansh | January 18, 2021 onwards | 4400 | 557 | 0.128 | 0.114 | 0.142 | 0.127 |
| No physical activity | French | January 18, 2021 onwards | 1341 | 294 | 0.221 | 0.192 | 0.254 | 0.219 |
| No physical activity | Italian | January 18, 2021 onwards | 978 | 187 | 0.194 | 0.161 | 0.232 | 0.191 |
| Health care use | German/Romansh | March 16, 2020 to May 10, 2020 | 4383 | 578 | 0.133 | 0.119 | 0.147 | 0.132 |
| Health care use | French | March 16, 2020 to May 10, 2020 | 1460 | 171 | 0.116 | 0.095 | 0.140 | 0.117 |
| Health care use | Italian | March 16, 2020 to May 10, 2020 | 989 | 103 | 0.105 | 0.081 | 0.135 | 0.104 |
| Health care use | German/Romansh | May 11, 2020 to July 5, 2020 | 3118 | 637 | 0.215 | 0.197 | 0.235 | 0.204 |
| Health care use | French | May 11, 2020 to July 5, 2020 | 1018 | 205 | 0.206 | 0.176 | 0.240 | 0.201 |
| Health care use | Italian | May 11, 2020 to July 5, 2020 | 675 | 135 | 0.210 | 0.173 | 0.252 | 0.200 |
| Health care use | German/Romansh | July 6, 2020 to October 18, 2020 | 2973 | 610 | 0.212 | 0.194 | 0.231 | 0.205 |
| Health care use | French | July 6, 2020 to October 18, 2020 | 949 | 181 | 0.198 | 0.167 | 0.232 | 0.191 |
| Health care use | Italian | July 6, 2020 to October 18, 2020 | 629 | 139 | 0.230 | 0.192 | 0.273 | 0.221 |
| Health care use | German/Romansh | October 19, 2020 to January 17, 2021 | 4433 | 1152 | 0.267 | 0.250 | 0.283 | 0.260 |
| Health care use | French | October 19, 2020 to January 17, 2021 | 1421 | 338 | 0.245 | 0.218 | 0.274 | 0.238 |
| Health care use | Italian | October 19, 2020 to January 17, 2021 | 1004 | 255 | 0.265 | 0.232 | 0.301 | 0.254 |
| Health care use | German/Romansh | January 18, 2021 onwards | 4400 | 1100 | 0.254 | 0.238 | 0.271 | 0.250 |
| Health care use | French | January 18, 2021 onwards | 1341 | 357 | 0.267 | 0.238 | 0.298 | 0.266 |
| Health care use | Italian | January 18, 2021 onwards | 978 | 262 | 0.272 | 0.238 | 0.309 | 0.268 |
| Health care non-use | German/Romansh | March 16, 2020 to May 10, 2020 | 4383 | 606 | 0.141 | 0.127 | 0.157 | 0.138 |
| Health care non-use | French | March 16, 2020 to May 10, 2020 | 1460 | 187 | 0.129 | 0.106 | 0.155 | 0.128 |
| Health care non-use | Italian | March 16, 2020 to May 10, 2020 | 989 | 160 | 0.163 | 0.132 | 0.199 | 0.162 |
| Health care non-use | German/Romansh | May 11, 2020 to July 5, 2020 | 3118 | 87 | 0.029 | 0.022 | 0.038 | 0.028 |
| Health care non-use | French | May 11, 2020 to July 5, 2020 | 1018 | 30 | 0.030 | 0.020 | 0.046 | 0.029 |
| Health care non-use | Italian | May 11, 2020 to July 5, 2020 | 675 | 34 | 0.051 | 0.034 | 0.075 | 0.050 |
| Health care non-use | German/Romansh | July 6, 2020 to October 18, 2020 | 2973 | 23 | 0.008 | 0.005 | 0.013 | 0.008 |
| Health care non-use | French | July 6, 2020 to October 18, 2020 | 949 | 10 | 0.010 | 0.004 | 0.022 | 0.011 |
| Health care non-use | Italian | July 6, 2020 to October 18, 2020 | 629 | 7 | 0.011 | 0.004 | 0.032 | 0.011 |
| Health care non-use | German/Romansh | October 19, 2020 to January 17, 2021 | 4433 | 82 | 0.019 | 0.015 | 0.025 | 0.018 |
| Health care non-use | French | October 19, 2020 to January 17, 2021 | 1421 | 31 | 0.024 | 0.017 | 0.036 | 0.022 |
| Health care non-use | Italian | October 19, 2020 to January 17, 2021 | 1004 | 38 | 0.040 | 0.028 | 0.057 | 0.038 |
| Health care non-use | German/Romansh | January 18, 2021 onwards | 4400 | 58 | 0.013 | 0.010 | 0.018 | 0.013 |
| Health care non-use | French | January 18, 2021 onwards | 1341 | 23 | 0.017 | 0.010 | 0.027 | 0.017 |
| Health care non-use | Italian | January 18, 2021 onwards | 978 | 27 | 0.027 | 0.016 | 0.043 | 0.028 |
| COVID-19 related health care use | German/Romansh | March 16, 2020 to May 10, 2020 | 4383 | 95 | 0.020 | 0.015 | 0.025 | 0.022 |
| COVID-19 related health care use | French | March 16, 2020 to May 10, 2020 | 1460 | 54 | 0.036 | 0.026 | 0.050 | 0.037 |
| COVID-19 related health care use | Italian | March 16, 2020 to May 10, 2020 | 989 | 28 | 0.026 | 0.017 | 0.038 | 0.028 |
| COVID-19 related health care use | German/Romansh | May 11, 2020 to July 5, 2020 | 3118 | 37 | 0.012 | 0.008 | 0.017 | 0.012 |
| COVID-19 related health care use | French | May 11, 2020 to July 5, 2020 | 1018 | 17 | 0.016 | 0.010 | 0.026 | 0.017 |
| COVID-19 related health care use | Italian | May 11, 2020 to July 5, 2020 | 675 | 8 | 0.013 | 0.006 | 0.026 | 0.012 |
| COVID-19 related health care use | German/Romansh | July 6, 2020 to October 18, 2020 | 2973 | 51 | 0.016 | 0.012 | 0.022 | 0.017 |
| COVID-19 related health care use | French | July 6, 2020 to October 18, 2020 | 949 | 25 | 0.026 | 0.017 | 0.039 | 0.026 |
| COVID-19 related health care use | Italian | July 6, 2020 to October 18, 2020 | 629 | 15 | 0.024 | 0.015 | 0.039 | 0.024 |
| COVID-19 related health care use | German/Romansh | October 19, 2020 to January 17, 2021 | 4433 | 133 | 0.030 | 0.025 | 0.036 | 0.030 |
| COVID-19 related health care use | French | October 19, 2020 to January 17, 2021 | 1421 | 43 | 0.032 | 0.024 | 0.043 | 0.030 |
| COVID-19 related health care use | Italian | October 19, 2020 to January 17, 2021 | 1004 | 26 | 0.027 | 0.019 | 0.040 | 0.026 |
| COVID-19 related health care use | German/Romansh | January 18, 2021 onwards | 4400 | 109 | 0.025 | 0.020 | 0.030 | 0.025 |
| COVID-19 related health care use | French | January 18, 2021 onwards | 1341 | 26 | 0.019 | 0.013 | 0.029 | 0.019 |
| COVID-19 related health care use | Italian | January 18, 2021 onwards | 978 | 27 | 0.027 | 0.019 | 0.039 | 0.028 |
| Adherence to physical distance | German/Romansh | March 16, 2020 to May 10, 2020 | 3091 | 1413 | 0.484 | 0.459 | 0.508 | 0.457 |
| Adherence to physical distance | French | March 16, 2020 to May 10, 2020 | 1023 | 600 | 0.606 | 0.565 | 0.645 | 0.587 |
| Adherence to physical distance | Italian | March 16, 2020 to May 10, 2020 | 692 | 479 | 0.705 | 0.658 | 0.748 | 0.692 |
| Adherence to physical distance | German/Romansh | May 11, 2020 to July 5, 2020 | 3118 | 885 | 0.295 | 0.273 | 0.318 | 0.284 |
| Adherence to physical distance | French | May 11, 2020 to July 5, 2020 | 1018 | 368 | 0.368 | 0.330 | 0.408 | 0.361 |
| Adherence to physical distance | Italian | May 11, 2020 to July 5, 2020 | 675 | 336 | 0.493 | 0.442 | 0.544 | 0.498 |
| Adherence to physical distance | German/Romansh | July 6, 2020 to October 18, 2020 | 2973 | 719 | 0.250 | 0.229 | 0.273 | 0.242 |
| Adherence to physical distance | French | July 6, 2020 to October 18, 2020 | 949 | 206 | 0.224 | 0.190 | 0.263 | 0.217 |
| Adherence to physical distance | Italian | July 6, 2020 to October 18, 2020 | 629 | 197 | 0.312 | 0.264 | 0.365 | 0.313 |
| Adherence to physical distance | German/Romansh | October 19, 2020 to January 17, 2021 | 4433 | 1850 | 0.418 | 0.398 | 0.438 | 0.417 |
| Adherence to physical distance | French | October 19, 2020 to January 17, 2021 | 1421 | 618 | 0.433 | 0.399 | 0.468 | 0.435 |
| Adherence to physical distance | Italian | October 19, 2020 to January 17, 2021 | 1004 | 574 | 0.560 | 0.518 | 0.601 | 0.572 |
| Adherence to physical distance | German/Romansh | January 18, 2021 onwards | 4400 | 1699 | 0.388 | 0.368 | 0.408 | 0.386 |
| Adherence to physical distance | French | January 18, 2021 onwards | 1341 | 552 | 0.414 | 0.378 | 0.450 | 0.412 |
| Adherence to physical distance | Italian | January 18, 2021 onwards | 978 | 535 | 0.543 | 0.499 | 0.587 | 0.547 |
| Wearing of face mask | German/Romansh | March 16, 2020 to May 10, 2020 | 3091 | 38 | 0.013 | 0.009 | 0.018 | 0.012 |
| Wearing of face mask | French | March 16, 2020 to May 10, 2020 | 1023 | 62 | 0.062 | 0.045 | 0.084 | 0.061 |
| Wearing of face mask | Italian | March 16, 2020 to May 10, 2020 | 692 | 164 | 0.242 | 0.201 | 0.289 | 0.237 |
| Wearing of face mask | German/Romansh | May 11, 2020 to July 5, 2020 | 3118 | 75 | 0.025 | 0.019 | 0.034 | 0.024 |
| Wearing of face mask | French | May 11, 2020 to July 5, 2020 | 1018 | 82 | 0.083 | 0.062 | 0.111 | 0.081 |
| Wearing of face mask | Italian | May 11, 2020 to July 5, 2020 | 675 | 164 | 0.247 | 0.204 | 0.296 | 0.243 |
| Wearing of face mask | German/Romansh | July 6, 2020 to October 18, 2020 | 2973 | 428 | 0.149 | 0.133 | 0.167 | 0.144 |
| Wearing of face mask | French | July 6, 2020 to October 18, 2020 | 949 | 283 | 0.308 | 0.272 | 0.347 | 0.298 |
| Wearing of face mask | Italian | July 6, 2020 to October 18, 2020 | 629 | 191 | 0.309 | 0.260 | 0.364 | 0.304 |
| Wearing of face mask | German/Romansh | October 19, 2020 to January 17, 2021 | 4433 | 3570 | 0.771 | 0.756 | 0.786 | 0.805 |
| Wearing of face mask | French | October 19, 2020 to January 17, 2021 | 1421 | 1156 | 0.793 | 0.766 | 0.817 | 0.814 |
| Wearing of face mask | Italian | October 19, 2020 to January 17, 2021 | 1004 | 878 | 0.860 | 0.828 | 0.887 | 0.875 |
| Wearing of face mask | German/Romansh | January 18, 2021 onwards | 4400 | 3676 | 0.838 | 0.823 | 0.852 | 0.835 |
| Wearing of face mask | French | January 18, 2021 onwards | 1341 | 1113 | 0.834 | 0.806 | 0.858 | 0.830 |
| Wearing of face mask | Italian | January 18, 2021 onwards | 978 | 851 | 0.868 | 0.836 | 0.895 | 0.870 |
| Avoidance of private appointments | German/Romansh | March 16, 2020 to May 10, 2020 | 3091 | 1756 | 0.584 | 0.561 | 0.607 | 0.568 |
| Avoidance of private appointments | French | March 16, 2020 to May 10, 2020 | 1023 | 630 | 0.628 | 0.590 | 0.665 | 0.616 |
| Avoidance of private appointments | Italian | March 16, 2020 to May 10, 2020 | 692 | 553 | 0.800 | 0.761 | 0.835 | 0.799 |
| Avoidance of private appointments | German/Romansh | May 11, 2020 to July 5, 2020 | 3118 | 722 | 0.234 | 0.216 | 0.253 | 0.232 |
| Avoidance of private appointments | French | May 11, 2020 to July 5, 2020 | 1018 | 234 | 0.227 | 0.198 | 0.259 | 0.230 |
| Avoidance of private appointments | Italian | May 11, 2020 to July 5, 2020 | 675 | 235 | 0.335 | 0.291 | 0.382 | 0.348 |
| Avoidance of private appointments | German/Romansh | July 6, 2020 to October 18, 2020 | 2973 | 249 | 0.086 | 0.074 | 0.100 | 0.084 |
| Avoidance of private appointments | French | July 6, 2020 to October 18, 2020 | 949 | 69 | 0.076 | 0.057 | 0.101 | 0.073 |
| Avoidance of private appointments | Italian | July 6, 2020 to October 18, 2020 | 629 | 71 | 0.110 | 0.081 | 0.146 | 0.113 |
| Avoidance of private appointments | German/Romansh | October 19, 2020 to January 17, 2021 | 4433 | 1162 | 0.259 | 0.243 | 0.276 | 0.262 |
| Avoidance of private appointments | French | October 19, 2020 to January 17, 2021 | 1421 | 343 | 0.252 | 0.223 | 0.283 | 0.241 |
| Avoidance of private appointments | Italian | October 19, 2020 to January 17, 2021 | 1004 | 375 | 0.367 | 0.329 | 0.406 | 0.374 |
| Avoidance of private appointments | German/Romansh | January 18, 2021 onwards | 4400 | 822 | 0.186 | 0.171 | 0.201 | 0.187 |
| Avoidance of private appointments | French | January 18, 2021 onwards | 1341 | 208 | 0.153 | 0.128 | 0.181 | 0.155 |
| Avoidance of private appointments | Italian | January 18, 2021 onwards | 978 | 276 | 0.277 | 0.240 | 0.318 | 0.282 |
| Non-use of public transport | German/Romansh | March 16, 2020 to May 10, 2020 | 3091 | 2282 | 0.742 | 0.719 | 0.763 | 0.738 |
| Non-use of public transport | French | March 16, 2020 to May 10, 2020 | 1023 | 860 | 0.848 | 0.814 | 0.876 | 0.841 |
| Non-use of public transport | Italian | March 16, 2020 to May 10, 2020 | 692 | 638 | 0.918 | 0.887 | 0.941 | 0.922 |
| Non-use of public transport | German/Romansh | May 11, 2020 to July 5, 2020 | 3118 | 1824 | 0.583 | 0.558 | 0.608 | 0.585 |
| Non-use of public transport | French | May 11, 2020 to July 5, 2020 | 1018 | 692 | 0.683 | 0.642 | 0.721 | 0.680 |
| Non-use of public transport | Italian | May 11, 2020 to July 5, 2020 | 675 | 514 | 0.761 | 0.714 | 0.802 | 0.761 |
| Non-use of public transport | German/Romansh | July 6, 2020 to October 18, 2020 | 2973 | 1210 | 0.407 | 0.382 | 0.432 | 0.407 |
| Non-use of public transport | French | July 6, 2020 to October 18, 2020 | 949 | 444 | 0.478 | 0.434 | 0.523 | 0.468 |
| Non-use of public transport | Italian | July 6, 2020 to October 18, 2020 | 629 | 370 | 0.589 | 0.533 | 0.643 | 0.588 |
| Non-use of public transport | German/Romansh | October 19, 2020 to January 17, 2021 | 4433 | 2320 | 0.521 | 0.499 | 0.542 | 0.523 |
| Non-use of public transport | French | October 19, 2020 to January 17, 2021 | 1421 | 799 | 0.565 | 0.528 | 0.600 | 0.562 |
| Non-use of public transport | Italian | October 19, 2020 to January 17, 2021 | 1004 | 708 | 0.708 | 0.666 | 0.746 | 0.705 |
| Non-use of public transport | German/Romansh | January 18, 2021 onwards | 4400 | 2216 | 0.498 | 0.477 | 0.520 | 0.504 |
| Non-use of public transport | French | January 18, 2021 onwards | 1341 | 663 | 0.493 | 0.454 | 0.532 | 0.494 |
| Non-use of public transport | Italian | January 18, 2021 onwards | 978 | 656 | 0.669 | 0.625 | 0.711 | 0.671 |
| Poor health status | Italian | March 16, 2020 to May 10, 2020 | 6832 | 67 | 0.009 | 0.006 | 0.013 | 0.010 |
| Poor health status | Italian | May 11, 2020 to July 5, 2020 | 4811 | 48 | 0.009 | 0.006 | 0.013 | 0.010 |
| Poor health status | Italian | July 6, 2020 to October 18, 2020 | 4551 | 69 | 0.014 | 0.010 | 0.020 | 0.015 |
| Poor health status | Italian | October 19, 2020 to January 17, 2021 | 6858 | 134 | 0.018 | 0.014 | 0.023 | 0.020 |
| Poor health status | Italian | January 18, 2021 onwards | 6719 | 135 | 0.020 | 0.015 | 0.025 | 0.020 |
| Poor quality of life | Italian | March 16, 2020 to May 10, 2020 | 6832 | 131 | 0.019 | 0.015 | 0.025 | 0.019 |
| Poor quality of life | Italian | May 11, 2020 to July 5, 2020 | 4811 | 78 | 0.017 | 0.012 | 0.023 | 0.016 |
| Poor quality of life | Italian | July 6, 2020 to October 18, 2020 | 4551 | 82 | 0.019 | 0.014 | 0.025 | 0.018 |
| Poor quality of life | Italian | October 19, 2020 to January 17, 2021 | 6858 | 255 | 0.037 | 0.032 | 0.044 | 0.037 |
| Poor quality of life | Italian | January 18, 2021 onwards | 6719 | 194 | 0.030 | 0.025 | 0.037 | 0.029 |
| Depressive mood | Italian | March 16, 2020 to May 10, 2020 | 6832 | 383 | 0.050 | 0.043 | 0.058 | 0.056 |
| Depressive mood | Italian | May 11, 2020 to July 5, 2020 | 4811 | 262 | 0.051 | 0.043 | 0.060 | 0.054 |
| Depressive mood | Italian | July 6, 2020 to October 18, 2020 | 4551 | 208 | 0.044 | 0.036 | 0.053 | 0.046 |
| Depressive mood | Italian | October 19, 2020 to January 17, 2021 | 6858 | 522 | 0.070 | 0.062 | 0.078 | 0.076 |
| Depressive mood | Italian | January 18, 2021 onwards | 6719 | 455 | 0.065 | 0.057 | 0.074 | 0.068 |
| Lack of energy | Italian | March 16, 2020 to May 10, 2020 | 6832 | 974 | 0.139 | 0.126 | 0.152 | 0.143 |
| Lack of energy | Italian | May 11, 2020 to July 5, 2020 | 4811 | 614 | 0.124 | 0.111 | 0.138 | 0.128 |
| Lack of energy | Italian | July 6, 2020 to October 18, 2020 | 4551 | 543 | 0.118 | 0.105 | 0.132 | 0.119 |
| Lack of energy | Italian | October 19, 2020 to January 17, 2021 | 6858 | 1251 | 0.177 | 0.165 | 0.191 | 0.182 |
| Lack of energy | Italian | January 18, 2021 onwards | 6719 | 1141 | 0.167 | 0.155 | 0.181 | 0.170 |
| Fear of loosing employment | Italian | March 16, 2020 to May 10, 2020 | 4805 | 454 | 0.090 | 0.077 | 0.105 | 0.094 |
| Fear of loosing employment | Italian | May 11, 2020 to July 5, 2020 | 3356 | 272 | 0.078 | 0.065 | 0.093 | 0.081 |
| Fear of loosing employment | Italian | July 6, 2020 to October 18, 2020 | 3205 | 260 | 0.079 | 0.065 | 0.094 | 0.081 |
| Fear of loosing employment | Italian | October 19, 2020 to January 17, 2021 | 4825 | 420 | 0.082 | 0.071 | 0.094 | 0.087 |
| Fear of loosing employment | Italian | January 18, 2021 onwards | 4632 | 334 | 0.066 | 0.056 | 0.077 | 0.072 |
| Feelings of loneliness | Italian | March 16, 2020 to May 10, 2020 | 6832 | 140 | 0.020 | 0.015 | 0.026 | 0.020 |
| Feelings of loneliness | Italian | May 11, 2020 to July 5, 2020 | 4811 | 75 | 0.015 | 0.011 | 0.022 | 0.016 |
| Feelings of loneliness | Italian | July 6, 2020 to October 18, 2020 | 4551 | 58 | 0.013 | 0.009 | 0.019 | 0.013 |
| Feelings of loneliness | Italian | October 19, 2020 to January 17, 2021 | 6858 | 148 | 0.021 | 0.017 | 0.027 | 0.022 |
| Feelings of loneliness | Italian | January 18, 2021 onwards | 6719 | 150 | 0.023 | 0.018 | 0.029 | 0.022 |
| Population 65 years or older: Feelings of social isolation | Italian | March 16, 2020 to May 10, 2020 | 736 | 55 | 0.077 | 0.055 | 0.106 | 0.075 |
| Population 65 years or older: Feelings of social isolation | Italian | May 11, 2020 to July 5, 2020 | 778 | 38 | 0.059 | 0.037 | 0.092 | 0.049 |
| Population 65 years or older: Feelings of social isolation | Italian | July 6, 2020 to October 18, 2020 | 757 | 21 | 0.032 | 0.017 | 0.059 | 0.028 |
| Population 65 years or older: Feelings of social isolation | Italian | October 19, 2020 to January 17, 2021 | 1162 | 76 | 0.064 | 0.046 | 0.087 | 0.065 |
| Population 65 years or older: Feelings of social isolation | Italian | January 18, 2021 onwards | 1228 | 70 | 0.056 | 0.039 | 0.081 | 0.057 |
| No physical activity | Italian | March 16, 2020 to May 10, 2020 | 6832 | 1103 | 0.152 | 0.138 | 0.166 | 0.161 |
| No physical activity | Italian | May 11, 2020 to July 5, 2020 | 4811 | 606 | 0.117 | 0.105 | 0.131 | 0.126 |
| No physical activity | Italian | July 6, 2020 to October 18, 2020 | 4551 | 584 | 0.122 | 0.109 | 0.136 | 0.128 |
| No physical activity | Italian | October 19, 2020 to January 17, 2021 | 6858 | 1287 | 0.172 | 0.160 | 0.185 | 0.188 |
| No physical activity | Italian | January 18, 2021 onwards | 6719 | 1038 | 0.153 | 0.141 | 0.166 | 0.154 |
| Health care use | Italian | March 16, 2020 to May 10, 2020 | 6832 | 852 | 0.127 | 0.116 | 0.139 | 0.125 |
| Health care use | Italian | May 11, 2020 to July 5, 2020 | 4811 | 977 | 0.213 | 0.197 | 0.229 | 0.203 |
| Health care use | Italian | July 6, 2020 to October 18, 2020 | 4551 | 930 | 0.209 | 0.194 | 0.225 | 0.204 |
| Health care use | Italian | October 19, 2020 to January 17, 2021 | 6858 | 1745 | 0.261 | 0.248 | 0.275 | 0.254 |
| Health care use | Italian | January 18, 2021 onwards | 6719 | 1719 | 0.258 | 0.245 | 0.272 | 0.256 |
| Health care non-use | Italian | March 16, 2020 to May 10, 2020 | 6832 | 953 | 0.139 | 0.128 | 0.152 | 0.139 |
| Health care non-use | Italian | May 11, 2020 to July 5, 2020 | 4811 | 151 | 0.030 | 0.024 | 0.037 | 0.031 |
| Health care non-use | Italian | July 6, 2020 to October 18, 2020 | 4551 | 40 | 0.009 | 0.006 | 0.013 | 0.009 |
| Health care non-use | Italian | October 19, 2020 to January 17, 2021 | 6858 | 151 | 0.021 | 0.018 | 0.026 | 0.022 |
| Health care non-use | Italian | January 18, 2021 onwards | 6719 | 108 | 0.015 | 0.011 | 0.019 | 0.016 |
| COVID-19 related health care use | Italian | March 16, 2020 to May 10, 2020 | 6832 | 177 | 0.024 | 0.020 | 0.029 | 0.026 |
| COVID-19 related health care use | Italian | May 11, 2020 to July 5, 2020 | 4811 | 62 | 0.013 | 0.010 | 0.017 | 0.013 |
| COVID-19 related health care use | Italian | July 6, 2020 to October 18, 2020 | 4551 | 91 | 0.019 | 0.015 | 0.024 | 0.020 |
| COVID-19 related health care use | Italian | October 19, 2020 to January 17, 2021 | 6858 | 202 | 0.030 | 0.026 | 0.035 | 0.029 |
| COVID-19 related health care use | Italian | January 18, 2021 onwards | 6719 | 162 | 0.024 | 0.020 | 0.028 | 0.024 |
| Adherence to physical distance | Italian | March 16, 2020 to May 10, 2020 | 4806 | 2492 | 0.523 | 0.503 | 0.543 | 0.519 |
| Adherence to physical distance | Italian | May 11, 2020 to July 5, 2020 | 4811 | 1589 | 0.322 | 0.303 | 0.341 | 0.330 |
| Adherence to physical distance | Italian | July 6, 2020 to October 18, 2020 | 4551 | 1122 | 0.247 | 0.229 | 0.265 | 0.247 |
| Adherence to physical distance | Italian | October 19, 2020 to January 17, 2021 | 6858 | 3042 | 0.428 | 0.411 | 0.444 | 0.444 |
| Adherence to physical distance | Italian | January 18, 2021 onwards | 6719 | 2786 | 0.401 | 0.384 | 0.418 | 0.415 |
| Wearing of face mask | Italian | March 16, 2020 to May 10, 2020 | 4806 | 264 | 0.034 | 0.029 | 0.041 | 0.055 |
| Wearing of face mask | Italian | May 11, 2020 to July 5, 2020 | 4811 | 321 | 0.049 | 0.041 | 0.058 | 0.067 |
| Wearing of face mask | Italian | July 6, 2020 to October 18, 2020 | 4551 | 902 | 0.194 | 0.179 | 0.211 | 0.198 |
| Wearing of face mask | Italian | October 19, 2020 to January 17, 2021 | 6858 | 5604 | 0.780 | 0.767 | 0.793 | 0.817 |
| Wearing of face mask | Italian | January 18, 2021 onwards | 6719 | 5640 | 0.838 | 0.826 | 0.850 | 0.839 |
| Avoidance of private appointments | Italian | March 16, 2020 to May 10, 2020 | 4806 | 2939 | 0.604 | 0.585 | 0.623 | 0.612 |
| Avoidance of private appointments | Italian | May 11, 2020 to July 5, 2020 | 4811 | 1191 | 0.237 | 0.222 | 0.252 | 0.248 |
| Avoidance of private appointments | Italian | July 6, 2020 to October 18, 2020 | 4551 | 389 | 0.085 | 0.075 | 0.096 | 0.085 |
| Avoidance of private appointments | Italian | October 19, 2020 to January 17, 2021 | 6858 | 1880 | 0.262 | 0.248 | 0.277 | 0.274 |
| Avoidance of private appointments | Italian | January 18, 2021 onwards | 6719 | 1306 | 0.182 | 0.169 | 0.195 | 0.194 |
| Non-use of public transport | Italian | March 16, 2020 to May 10, 2020 | 4806 | 3780 | 0.775 | 0.757 | 0.792 | 0.787 |
| Non-use of public transport | Italian | May 11, 2020 to July 5, 2020 | 4811 | 3030 | 0.615 | 0.594 | 0.635 | 0.630 |
| Non-use of public transport | Italian | July 6, 2020 to October 18, 2020 | 4551 | 2024 | 0.432 | 0.411 | 0.453 | 0.445 |
| Non-use of public transport | Italian | October 19, 2020 to January 17, 2021 | 6858 | 3827 | 0.539 | 0.522 | 0.557 | 0.558 |
| Non-use of public transport | Italian | January 18, 2021 onwards | 6719 | 3535 | 0.505 | 0.486 | 0.523 | 0.526 |
