# Supplementary material for: Health and social behaviour through pandemic phases in Switzerland: Regional time-trends of the COVID-19 Social Monitor panel study
Source: PLoS One. 2021 Aug 25;16(8):e0256253. doi: 10.1371/journal.pone.0256253 (PMC8386858; doi:10.1371/journal.pone.0256253)
Supplement: S4 Table — (DOCX) [file pone.0256253.s004.docx]

**S5 Table.** p-values from likelihood ratio test for period effect, by language region.

| **Study outcome** | **Language region** | **p-value** |
| --- | --- | --- |
| Population 65 years or older: Feelings of social isolation | German/Romansh | <0.001 |
| Population 65 years or older: Feelings of social isolation | French | =0.005 |
| Population 65 years or older: Feelings of social isolation | Italian | =0.002 |
| No physical activity | German/Romansh | <0.001 |
| No physical activity | French | <0.001 |
| No physical activity | Italian | <0.001 |
| Adherence to physical distance | German/Romansh | <0.001 |
| Adherence to physical distance | French | <0.001 |
| Adherence to physical distance | Italian | <0.001 |
| Wearing of face mask | German/Romansh | <0.001 |
| Wearing of face mask | French | <0.001 |
| Wearing of face mask | Italian | <0.001 |
| Avoidance of private appointments | German/Romansh | <0.001 |
| Avoidance of private appointments | French | <0.001 |
| Avoidance of private appointments | Italian | <0.001 |
| Non-use of public transport | German/Romansh | <0.001 |
| Non-use of public transport | French | <0.001 |
| Non-use of public transport | Italian | <0.001 |
| Health care use | German/Romansh | <0.001 |
| Health care use | French | <0.001 |
| Health care use | Italian | <0.001 |
| Health care non-use | German/Romansh | <0.001 |
| Health care non-use | French | <0.001 |
| Health care non-use | Italian | <0.001 |
| COVID-19 related health care use | German/Romansh | <0.001 |
| COVID-19 related health care use | French | =0.012 |
| COVID-19 related health care use | Italian | =0.145 |
| Depressive mood | German/Romansh | <0.001 |
| Depressive mood | French | =0.012 |
| Depressive mood | Italian | <0.001 |
| Lack of energy | German/Romansh | <0.001 |
| Lack of energy | French | <0.001 |
| Lack of energy | Italian | <0.001 |
| Poor health status | German/Romansh | <0.001 |
| Poor health status | French | =0.004 |
| Poor health status | Italian | =0.019 |
| Feelings of loneliness | German/Romansh | =0.004 |
| Feelings of loneliness | French | =0.066 |
| Feelings of loneliness | Italian | =0.316 |
| Poor quality of life | German/Romansh | <0.001 |
| Poor quality of life | French | <0.001 |
| Poor quality of life | Italian | =0.06 |
| Fear of loosing employment | German/Romansh | <0.001 |
| Fear of loosing employment | French | <0.001 |
| Fear of loosing employment | Italian | =0.792 |
