# Supplementary material for: Health and social behaviour through pandemic phases in Switzerland: Regional time-trends of the COVID-19 Social Monitor panel study
Source: PLoS One. 2021 Aug 25;16(8):e0256253. doi: 10.1371/journal.pone.0256253 (PMC8386858; doi:10.1371/journal.pone.0256253)
Supplement: S5 Table — (DOCX) [file pone.0256253.s005.docx]

**S6 Table.** p-values from likelihood ratio test for interaction effect between language regions and mitigation periods from adjusted hierarchical logistic regression models.

| **Study outcome** | **p-value** |
| --- | --- |
| Poor health status | =0.998 |
| Poor quality of life | <0.001 |
| Depressive mood | =0.005 |
| Lack of energy | =0.97 |
| Fear of loosing employment | >0.999 |
| Feelings of loneliness | >0.999 |
| No physical activity | =0.036 |
| Health care use | =0.186 |
| Health care non-use | =0.361 |
| COVID-19 related health care use | =0.283 |
| Adherence to physical distance | <0.001 |
| Wearing of face mask | <0.001 |
| Avoidance of private appointments | <0.001 |
| Non-use of public transport | <0.001 |
| Population 65 years or older: Feelings of social isolation | =0.102 |
