# Supplementary material for: Health and social behaviour through pandemic phases in Switzerland: Regional time-trends of the COVID-19 Social Monitor panel study
Source: PLoS One. 2021 Aug 25;16(8):e0256253. doi: 10.1371/journal.pone.0256253 (PMC8386858; doi:10.1371/journal.pone.0256253)
Supplement: S6 Table — (DOCX) [file pone.0256253.s006.docx]

**S7 Table.** Time period effects from language region stratified adjusted hierarchical logistic regression models.

| **Study outcome** | **Language region** | **Period** | **Adjusted odds ratio** | **Lower 95% CI** | **Upper 95% CI** | **p-value** |
| --- | --- | --- | --- | --- | --- | --- |
| Poor health status | German/Romansh | March 16, 2020 to May 10, 2020 | 1.000 | NA | NA | NA |
| Poor health status | German/Romansh | May 11, 2020 to July 5, 2020 | 0.684 | 0.369 | 1.271 | 0.230 |
| Poor health status | German/Romansh | July 6, 2020 to October 18, 2020 | 1.348 | 0.772 | 2.354 | 0.294 |
| Poor health status | German/Romansh | October 19, 2020 to January 17, 2021 | 1.290 | 0.729 | 2.284 | 0.382 |
| Poor health status | German/Romansh | January 18, 2021 onwards | 1.709 | 0.958 | 3.048 | 0.069 |
| Poor health status | French | March 16, 2020 to May 10, 2020 | 1.000 | NA | NA | NA |
| Poor health status | French | May 11, 2020 to July 5, 2020 | 0.876 | 0.285 | 2.695 | 0.817 |
| Poor health status | French | July 6, 2020 to October 18, 2020 | 1.414 | 0.504 | 3.968 | 0.510 |
| Poor health status | French | October 19, 2020 to January 17, 2021 | 3.396 | 1.309 | 8.810 | 0.012 |
| Poor health status | French | January 18, 2021 onwards | 3.127 | 1.169 | 8.360 | 0.023 |
| Poor health status | Italian | March 16, 2020 to May 10, 2020 | 1.000 | NA | NA | NA |
| Poor health status | Italian | May 11, 2020 to July 5, 2020 | 0.891 | 0.241 | 3.303 | 0.863 |
| Poor health status | Italian | July 6, 2020 to October 18, 2020 | 1.937 | 0.553 | 6.786 | 0.301 |
| Poor health status | Italian | October 19, 2020 to January 17, 2021 | 3.692 | 1.102 | 12.366 | 0.034 |
| Poor health status | Italian | January 18, 2021 onwards | 1.551 | 0.417 | 5.768 | 0.512 |
| Poor quality of life | German/Romansh | March 16, 2020 to May 10, 2020 | 1.000 | NA | NA | NA |
| Poor quality of life | German/Romansh | May 11, 2020 to July 5, 2020 | 0.552 | 0.365 | 0.837 | 0.005 |
| Poor quality of life | German/Romansh | July 6, 2020 to October 18, 2020 | 0.508 | 0.335 | 0.773 | 0.002 |
| Poor quality of life | German/Romansh | October 19, 2020 to January 17, 2021 | 2.383 | 1.671 | 3.398 | <0.001 |
| Poor quality of life | German/Romansh | January 18, 2021 onwards | 1.246 | 0.849 | 1.828 | 0.261 |
| Poor quality of life | French | March 16, 2020 to May 10, 2020 | 1.000 | NA | NA | NA |
| Poor quality of life | French | May 11, 2020 to July 5, 2020 | 1.552 | 0.633 | 3.806 | 0.337 |
| Poor quality of life | French | July 6, 2020 to October 18, 2020 | 2.883 | 1.265 | 6.570 | 0.012 |
| Poor quality of life | French | October 19, 2020 to January 17, 2021 | 6.761 | 2.972 | 15.380 | <0.001 |
| Poor quality of life | French | January 18, 2021 onwards | 3.900 | 1.651 | 9.213 | 0.002 |
| Poor quality of life | Italian | March 16, 2020 to May 10, 2020 | 1.000 | NA | NA | NA |
| Poor quality of life | Italian | May 11, 2020 to July 5, 2020 | 0.158 | 0.034 | 0.742 | 0.019 |
| Poor quality of life | Italian | July 6, 2020 to October 18, 2020 | 0.107 | 0.017 | 0.689 | 0.019 |
| Poor quality of life | Italian | October 19, 2020 to January 17, 2021 | 0.960 | 0.355 | 2.596 | 0.935 |
| Poor quality of life | Italian | January 18, 2021 onwards | 0.362 | 0.106 | 1.232 | 0.104 |
| Depressive mood | German/Romansh | March 16, 2020 to May 10, 2020 | 1.000 | NA | NA | NA |
| Depressive mood | German/Romansh | May 11, 2020 to July 5, 2020 | 0.946 | 0.721 | 1.241 | 0.688 |
| Depressive mood | German/Romansh | July 6, 2020 to October 18, 2020 | 0.741 | 0.559 | 0.982 | 0.037 |
| Depressive mood | German/Romansh | October 19, 2020 to January 17, 2021 | 1.452 | 1.118 | 1.886 | 0.005 |
| Depressive mood | German/Romansh | January 18, 2021 onwards | 1.211 | 0.918 | 1.597 | 0.176 |
| Depressive mood | French | March 16, 2020 to May 10, 2020 | 1.000 | NA | NA | NA |
| Depressive mood | French | May 11, 2020 to July 5, 2020 | 0.955 | 0.618 | 1.475 | 0.834 |
| Depressive mood | French | July 6, 2020 to October 18, 2020 | 0.705 | 0.446 | 1.114 | 0.134 |
| Depressive mood | French | October 19, 2020 to January 17, 2021 | 1.387 | 0.909 | 2.118 | 0.129 |
| Depressive mood | French | January 18, 2021 onwards | 1.255 | 0.806 | 1.954 | 0.314 |
| Depressive mood | Italian | March 16, 2020 to May 10, 2020 | 1.000 | NA | NA | NA |
| Depressive mood | Italian | May 11, 2020 to July 5, 2020 | 0.606 | 0.364 | 1.008 | 0.054 |
| Depressive mood | Italian | July 6, 2020 to October 18, 2020 | 0.467 | 0.274 | 0.794 | 0.005 |
| Depressive mood | Italian | October 19, 2020 to January 17, 2021 | 1.723 | 1.076 | 2.757 | 0.023 |
| Depressive mood | Italian | January 18, 2021 onwards | 1.382 | 0.846 | 2.257 | 0.197 |
| Lack of energy | German/Romansh | March 16, 2020 to May 10, 2020 | 1.000 | NA | NA | NA |
| Lack of energy | German/Romansh | May 11, 2020 to July 5, 2020 | 0.493 | 0.365 | 0.666 | <0.001 |
| Lack of energy | German/Romansh | July 6, 2020 to October 18, 2020 | 0.319 | 0.211 | 0.481 | <0.001 |
| Lack of energy | German/Romansh | October 19, 2020 to January 17, 2021 | 0.851 | 0.710 | 1.021 | 0.083 |
| Lack of energy | German/Romansh | January 18, 2021 onwards | 0.738 | 0.615 | 0.887 | 0.001 |
| Lack of energy | French | March 16, 2020 to May 10, 2020 | 1.000 | NA | NA | NA |
| Lack of energy | French | May 11, 2020 to July 5, 2020 | 0.729 | 0.443 | 1.199 | 0.213 |
| Lack of energy | French | July 6, 2020 to October 18, 2020 | 0.338 | 0.168 | 0.681 | 0.002 |
| Lack of energy | French | October 19, 2020 to January 17, 2021 | 0.861 | 0.644 | 1.151 | 0.311 |
| Lack of energy | French | January 18, 2021 onwards | 0.673 | 0.498 | 0.910 | 0.010 |
| Lack of energy | Italian | March 16, 2020 to May 10, 2020 | 1.000 | NA | NA | NA |
| Lack of energy | Italian | May 11, 2020 to July 5, 2020 | 0.681 | 0.395 | 1.175 | 0.168 |
| Lack of energy | Italian | July 6, 2020 to October 18, 2020 | 0.624 | 0.296 | 1.318 | 0.216 |
| Lack of energy | Italian | October 19, 2020 to January 17, 2021 | 0.824 | 0.576 | 1.179 | 0.290 |
| Lack of energy | Italian | January 18, 2021 onwards | 0.728 | 0.505 | 1.049 | 0.088 |
| Fear of loosing employment | German/Romansh | March 16, 2020 to May 10, 2020 | 1.000 | NA | NA | NA |
| Fear of loosing employment | German/Romansh | May 11, 2020 to July 5, 2020 | 0.714 | 0.495 | 1.030 | 0.071 |
| Fear of loosing employment | German/Romansh | July 6, 2020 to October 18, 2020 | 0.925 | 0.644 | 1.330 | 0.674 |
| Fear of loosing employment | German/Romansh | October 19, 2020 to January 17, 2021 | 1.331 | 0.914 | 1.938 | 0.136 |
| Fear of loosing employment | German/Romansh | January 18, 2021 onwards | 0.521 | 0.340 | 0.798 | 0.003 |
| Fear of loosing employment | French | March 16, 2020 to May 10, 2020 | 1.000 | NA | NA | NA |
| Fear of loosing employment | French | May 11, 2020 to July 5, 2020 | 0.515 | 0.309 | 0.858 | 0.011 |
| Fear of loosing employment | French | July 6, 2020 to October 18, 2020 | 0.461 | 0.273 | 0.778 | 0.004 |
| Fear of loosing employment | French | October 19, 2020 to January 17, 2021 | 0.448 | 0.255 | 0.787 | 0.005 |
| Fear of loosing employment | French | January 18, 2021 onwards | 0.200 | 0.104 | 0.385 | <0.001 |
| Fear of loosing employment | Italian | March 16, 2020 to May 10, 2020 | 1.000 | NA | NA | NA |
| Fear of loosing employment | Italian | May 11, 2020 to July 5, 2020 | 0.789 | 0.368 | 1.692 | 0.543 |
| Fear of loosing employment | Italian | July 6, 2020 to October 18, 2020 | 0.696 | 0.328 | 1.475 | 0.344 |
| Fear of loosing employment | Italian | October 19, 2020 to January 17, 2021 | 0.746 | 0.346 | 1.608 | 0.454 |
| Fear of loosing employment | Italian | January 18, 2021 onwards | 0.857 | 0.386 | 1.900 | 0.704 |
| Feelings of loneliness | German/Romansh | March 16, 2020 to May 10, 2020 | 1.000 | NA | NA | NA |
| Feelings of loneliness | German/Romansh | May 11, 2020 to July 5, 2020 | 0.308 | 0.174 | 0.544 | <0.001 |
| Feelings of loneliness | German/Romansh | July 6, 2020 to October 18, 2020 | 0.201 | 0.110 | 0.369 | <0.001 |
| Feelings of loneliness | German/Romansh | October 19, 2020 to January 17, 2021 | 0.540 | 0.313 | 0.932 | 0.027 |
| Feelings of loneliness | German/Romansh | January 18, 2021 onwards | 0.547 | 0.311 | 0.964 | 0.037 |
| Feelings of loneliness | French | March 16, 2020 to May 10, 2020 | 1.000 | NA | NA | NA |
| Feelings of loneliness | French | May 11, 2020 to July 5, 2020 | 0.277 | 0.140 | 0.546 | <0.001 |
| Feelings of loneliness | French | July 6, 2020 to October 18, 2020 | 0.244 | 0.123 | 0.485 | <0.001 |
| Feelings of loneliness | French | October 19, 2020 to January 17, 2021 | 0.518 | 0.274 | 0.978 | 0.043 |
| Feelings of loneliness | French | January 18, 2021 onwards | 0.531 | 0.276 | 1.022 | 0.058 |
| Feelings of loneliness | Italian | March 16, 2020 to May 10, 2020 | 1.000 | NA | NA | NA |
| Feelings of loneliness | Italian | May 11, 2020 to July 5, 2020 | 0.409 | 0.151 | 1.108 | 0.079 |
| Feelings of loneliness | Italian | July 6, 2020 to October 18, 2020 | 0.216 | 0.068 | 0.684 | 0.009 |
| Feelings of loneliness | Italian | October 19, 2020 to January 17, 2021 | 0.708 | 0.271 | 1.853 | 0.482 |
| Feelings of loneliness | Italian | January 18, 2021 onwards | 0.807 | 0.307 | 2.118 | 0.662 |
| Population 65 years or older: Feelings of social isolation | German/Romansh | March 16, 2020 to May 10, 2020 | 1.000 | NA | NA | NA |
| Population 65 years or older: Feelings of social isolation | German/Romansh | May 11, 2020 to July 5, 2020 | 0.583 | 0.278 | 1.220 | 0.152 |
| Population 65 years or older: Feelings of social isolation | German/Romansh | July 6, 2020 to October 18, 2020 | 0.159 | 0.061 | 0.409 | <0.001 |
| Population 65 years or older: Feelings of social isolation | German/Romansh | October 19, 2020 to January 17, 2021 | 1.026 | 0.509 | 2.067 | 0.943 |
| Population 65 years or older: Feelings of social isolation | German/Romansh | January 18, 2021 onwards | 0.684 | 0.322 | 1.452 | 0.323 |
| Population 65 years or older: Feelings of social isolation | French | March 16, 2020 to May 10, 2020 | 1.000 | NA | NA | NA |
| Population 65 years or older: Feelings of social isolation | French | May 11, 2020 to July 5, 2020 | 0.531 | 0.200 | 1.411 | 0.205 |
| Population 65 years or older: Feelings of social isolation | French | July 6, 2020 to October 18, 2020 | 0.181 | 0.057 | 0.571 | 0.004 |
| Population 65 years or older: Feelings of social isolation | French | October 19, 2020 to January 17, 2021 | 0.317 | 0.111 | 0.903 | 0.032 |
| Population 65 years or older: Feelings of social isolation | French | January 18, 2021 onwards | 0.171 | 0.054 | 0.542 | 0.003 |
| Population 65 years or older: Feelings of social isolation | Italian | March 16, 2020 to May 10, 2020 | 1.000 | NA | NA | NA |
| Population 65 years or older: Feelings of social isolation | Italian | May 11, 2020 to July 5, 2020 | 0.010 | 0.0002 | 0.394 | 0.014 |
| Population 65 years or older: Feelings of social isolation | Italian | July 6, 2020 to October 18, 2020 | 0.124 | 0.017 | 0.885 | 0.037 |
| Population 65 years or older: Feelings of social isolation | Italian | October 19, 2020 to January 17, 2021 | 0.375 | 0.077 | 1.832 | 0.226 |
| Population 65 years or older: Feelings of social isolation | Italian | January 18, 2021 onwards | 0.298 | 0.053 | 1.670 | 0.169 |
| No physical activity | German/Romansh | March 16, 2020 to May 10, 2020 | 1.000 | NA | NA | NA |
| No physical activity | German/Romansh | May 11, 2020 to July 5, 2020 | 0.572 | 0.471 | 0.696 | <0.001 |
| No physical activity | German/Romansh | July 6, 2020 to October 18, 2020 | 0.633 | 0.522 | 0.768 | <0.001 |
| No physical activity | German/Romansh | October 19, 2020 to January 17, 2021 | 1.233 | 1.027 | 1.479 | 0.025 |
| No physical activity | German/Romansh | January 18, 2021 onwards | 0.968 | 0.797 | 1.176 | 0.744 |
| No physical activity | French | March 16, 2020 to May 10, 2020 | 1.000 | NA | NA | NA |
| No physical activity | French | May 11, 2020 to July 5, 2020 | 0.680 | 0.519 | 0.889 | 0.005 |
| No physical activity | French | July 6, 2020 to October 18, 2020 | 0.672 | 0.513 | 0.881 | 0.004 |
| No physical activity | French | October 19, 2020 to January 17, 2021 | 1.507 | 1.160 | 1.957 | 0.002 |
| No physical activity | French | January 18, 2021 onwards | 1.058 | 0.802 | 1.396 | 0.689 |
| No physical activity | Italian | March 16, 2020 to May 10, 2020 | 1.000 | NA | NA | NA |
| No physical activity | Italian | May 11, 2020 to July 5, 2020 | 0.861 | 0.606 | 1.222 | 0.402 |
| No physical activity | Italian | July 6, 2020 to October 18, 2020 | 0.949 | 0.668 | 1.347 | 0.768 |
| No physical activity | Italian | October 19, 2020 to January 17, 2021 | 1.728 | 1.222 | 2.445 | 0.002 |
| No physical activity | Italian | January 18, 2021 onwards | 0.761 | 0.522 | 1.111 | 0.157 |
| Health care use | German/Romansh | March 16, 2020 to May 10, 2020 | 1.000 | NA | NA | NA |
| Health care use | German/Romansh | May 11, 2020 to July 5, 2020 | 2.086 | 1.810 | 2.404 | <0.001 |
| Health care use | German/Romansh | July 6, 2020 to October 18, 2020 | 2.060 | 1.787 | 2.375 | <0.001 |
| Health care use | German/Romansh | October 19, 2020 to January 17, 2021 | 3.065 | 2.666 | 3.524 | <0.001 |
| Health care use | German/Romansh | January 18, 2021 onwards | 2.779 | 2.408 | 3.207 | <0.001 |
| Health care use | French | March 16, 2020 to May 10, 2020 | 1.000 | NA | NA | NA |
| Health care use | French | May 11, 2020 to July 5, 2020 | 2.350 | 1.822 | 3.030 | <0.001 |
| Health care use | French | July 6, 2020 to October 18, 2020 | 2.030 | 1.570 | 2.624 | <0.001 |
| Health care use | French | October 19, 2020 to January 17, 2021 | 2.943 | 2.289 | 3.783 | <0.001 |
| Health care use | French | January 18, 2021 onwards | 3.398 | 2.634 | 4.385 | <0.001 |
| Health care use | Italian | March 16, 2020 to May 10, 2020 | 1.000 | NA | NA | NA |
| Health care use | Italian | May 11, 2020 to July 5, 2020 | 2.562 | 1.872 | 3.507 | <0.001 |
| Health care use | Italian | July 6, 2020 to October 18, 2020 | 3.003 | 2.197 | 4.105 | <0.001 |
| Health care use | Italian | October 19, 2020 to January 17, 2021 | 3.869 | 2.844 | 5.264 | <0.001 |
| Health care use | Italian | January 18, 2021 onwards | 4.171 | 3.050 | 5.704 | <0.001 |
| Health care non-use | German/Romansh | March 16, 2020 to May 10, 2020 | 1.000 | NA | NA | NA |
| Health care non-use | German/Romansh | May 11, 2020 to July 5, 2020 | 0.101 | 0.077 | 0.133 | <0.001 |
| Health care non-use | German/Romansh | July 6, 2020 to October 18, 2020 | 0.022 | 0.014 | 0.035 | <0.001 |
| Health care non-use | German/Romansh | October 19, 2020 to January 17, 2021 | 0.073 | 0.054 | 0.098 | <0.001 |
| Health care non-use | German/Romansh | January 18, 2021 onwards | 0.047 | 0.033 | 0.066 | <0.001 |
| Health care non-use | French | March 16, 2020 to May 10, 2020 | 1.000 | NA | NA | NA |
| Health care non-use | French | May 11, 2020 to July 5, 2020 | 0.127 | 0.080 | 0.201 | <0.001 |
| Health care non-use | French | July 6, 2020 to October 18, 2020 | 0.039 | 0.020 | 0.078 | <0.001 |
| Health care non-use | French | October 19, 2020 to January 17, 2021 | 0.118 | 0.074 | 0.189 | <0.001 |
| Health care non-use | French | January 18, 2021 onwards | 0.074 | 0.042 | 0.128 | <0.001 |
| Health care non-use | Italian | March 16, 2020 to May 10, 2020 | 1.000 | NA | NA | NA |
| Health care non-use | Italian | May 11, 2020 to July 5, 2020 | 0.168 | 0.107 | 0.264 | <0.001 |
| Health care non-use | Italian | July 6, 2020 to October 18, 2020 | 0.021 | 0.009 | 0.050 | <0.001 |
| Health care non-use | Italian | October 19, 2020 to January 17, 2021 | 0.124 | 0.077 | 0.201 | <0.001 |
| Health care non-use | Italian | January 18, 2021 onwards | 0.070 | 0.040 | 0.124 | <0.001 |
| COVID-19 related health care use | German/Romansh | March 16, 2020 to May 10, 2020 | 1.000 | NA | NA | NA |
| COVID-19 related health care use | German/Romansh | May 11, 2020 to July 5, 2020 | 0.501 | 0.333 | 0.755 | 0.001 |
| COVID-19 related health care use | German/Romansh | July 6, 2020 to October 18, 2020 | 0.809 | 0.563 | 1.162 | 0.252 |
| COVID-19 related health care use | German/Romansh | October 19, 2020 to January 17, 2021 | 1.557 | 1.140 | 2.128 | 0.005 |
| COVID-19 related health care use | German/Romansh | January 18, 2021 onwards | 1.279 | 0.921 | 1.777 | 0.142 |
| COVID-19 related health care use | French | March 16, 2020 to May 10, 2020 | 1.000 | NA | NA | NA |
| COVID-19 related health care use | French | May 11, 2020 to July 5, 2020 | 0.385 | 0.218 | 0.682 | 0.001 |
| COVID-19 related health care use | French | July 6, 2020 to October 18, 2020 | 0.651 | 0.398 | 1.064 | 0.087 |
| COVID-19 related health care use | French | October 19, 2020 to January 17, 2021 | 0.842 | 0.536 | 1.322 | 0.454 |
| COVID-19 related health care use | French | January 18, 2021 onwards | 0.523 | 0.311 | 0.878 | 0.014 |
| COVID-19 related health care use | Italian | March 16, 2020 to May 10, 2020 | 1.000 | NA | NA | NA |
| COVID-19 related health care use | Italian | May 11, 2020 to July 5, 2020 | 0.377 | 0.171 | 0.834 | 0.016 |
| COVID-19 related health care use | Italian | July 6, 2020 to October 18, 2020 | 0.787 | 0.422 | 1.467 | 0.451 |
| COVID-19 related health care use | Italian | October 19, 2020 to January 17, 2021 | 0.887 | 0.495 | 1.590 | 0.688 |
| COVID-19 related health care use | Italian | January 18, 2021 onwards | 0.960 | 0.543 | 1.696 | 0.887 |
| Adherence to physical distance | German/Romansh | March 16, 2020 to May 10, 2020 | 1.000 | NA | NA | NA |
| Adherence to physical distance | German/Romansh | May 11, 2020 to July 5, 2020 | 0.248 | 0.215 | 0.286 | <0.001 |
| Adherence to physical distance | German/Romansh | July 6, 2020 to October 18, 2020 | 0.168 | 0.145 | 0.195 | <0.001 |
| Adherence to physical distance | German/Romansh | October 19, 2020 to January 17, 2021 | 0.637 | 0.555 | 0.731 | <0.001 |
| Adherence to physical distance | German/Romansh | January 18, 2021 onwards | 0.465 | 0.402 | 0.537 | <0.001 |
| Adherence to physical distance | French | March 16, 2020 to May 10, 2020 | 1.000 | NA | NA | NA |
| Adherence to physical distance | French | May 11, 2020 to July 5, 2020 | 0.219 | 0.174 | 0.277 | <0.001 |
| Adherence to physical distance | French | July 6, 2020 to October 18, 2020 | 0.064 | 0.049 | 0.084 | <0.001 |
| Adherence to physical distance | French | October 19, 2020 to January 17, 2021 | 0.308 | 0.243 | 0.390 | <0.001 |
| Adherence to physical distance | French | January 18, 2021 onwards | 0.246 | 0.192 | 0.315 | <0.001 |
| Adherence to physical distance | Italian | March 16, 2020 to May 10, 2020 | 1.000 | NA | NA | NA |
| Adherence to physical distance | Italian | May 11, 2020 to July 5, 2020 | 0.211 | 0.156 | 0.287 | <0.001 |
| Adherence to physical distance | Italian | July 6, 2020 to October 18, 2020 | 0.053 | 0.038 | 0.075 | <0.001 |
| Adherence to physical distance | Italian | October 19, 2020 to January 17, 2021 | 0.302 | 0.221 | 0.412 | <0.001 |
| Adherence to physical distance | Italian | January 18, 2021 onwards | 0.242 | 0.175 | 0.334 | <0.001 |
| Wearing of face mask | German/Romansh | March 16, 2020 to May 10, 2020 | 1.000 | NA | NA | NA |
| Wearing of face mask | German/Romansh | May 11, 2020 to July 5, 2020 | 1.817 | 1.224 | 2.696 | 0.003 |
| Wearing of face mask | German/Romansh | July 6, 2020 to October 18, 2020 | 22.441 | 15.876 | 31.721 | <0.001 |
| Wearing of face mask | German/Romansh | October 19, 2020 to January 17, 2021 | 2233.005 | 1512.211 | 3297.363 | <0.001 |
| Wearing of face mask | German/Romansh | January 18, 2021 onwards | 3372.700 | 2268.232 | 5014.967 | <0.001 |
| Wearing of face mask | French | March 16, 2020 to May 10, 2020 | 1.000 | NA | NA | NA |
| Wearing of face mask | French | May 11, 2020 to July 5, 2020 | 1.374 | 0.929 | 2.033 | 0.112 |
| Wearing of face mask | French | July 6, 2020 to October 18, 2020 | 14.621 | 10.177 | 21.005 | <0.001 |
| Wearing of face mask | French | October 19, 2020 to January 17, 2021 | 434.969 | 281.858 | 671.254 | <0.001 |
| Wearing of face mask | French | January 18, 2021 onwards | 479.376 | 309.319 | 742.929 | <0.001 |
| Wearing of face mask | Italian | March 16, 2020 to May 10, 2020 | 1.000 | NA | NA | NA |
| Wearing of face mask | Italian | May 11, 2020 to July 5, 2020 | 0.915 | 0.662 | 1.263 | 0.588 |
| Wearing of face mask | Italian | July 6, 2020 to October 18, 2020 | 1.658 | 1.209 | 2.275 | 0.002 |
| Wearing of face mask | Italian | October 19, 2020 to January 17, 2021 | 134.522 | 86.187 | 209.964 | <0.001 |
| Wearing of face mask | Italian | January 18, 2021 onwards | 130.474 | 83.251 | 204.482 | <0.001 |
| Avoidance of private appointments | German/Romansh | March 16, 2020 to May 10, 2020 | 1.000 | NA | NA | NA |
| Avoidance of private appointments | German/Romansh | May 11, 2020 to July 5, 2020 | 0.112 | 0.097 | 0.129 | <0.001 |
| Avoidance of private appointments | German/Romansh | July 6, 2020 to October 18, 2020 | 0.020 | 0.017 | 0.025 | <0.001 |
| Avoidance of private appointments | German/Romansh | October 19, 2020 to January 17, 2021 | 0.128 | 0.111 | 0.148 | <0.001 |
| Avoidance of private appointments | German/Romansh | January 18, 2021 onwards | 0.065 | 0.056 | 0.076 | <0.001 |
| Avoidance of private appointments | French | March 16, 2020 to May 10, 2020 | 1.000 | NA | NA | NA |
| Avoidance of private appointments | French | May 11, 2020 to July 5, 2020 | 0.070 | 0.054 | 0.091 | <0.001 |
| Avoidance of private appointments | French | July 6, 2020 to October 18, 2020 | 0.012 | 0.008 | 0.017 | <0.001 |
| Avoidance of private appointments | French | October 19, 2020 to January 17, 2021 | 0.092 | 0.071 | 0.118 | <0.001 |
| Avoidance of private appointments | French | January 18, 2021 onwards | 0.038 | 0.028 | 0.051 | <0.001 |
| Avoidance of private appointments | Italian | March 16, 2020 to May 10, 2020 | 1.000 | NA | NA | NA |
| Avoidance of private appointments | Italian | May 11, 2020 to July 5, 2020 | 0.051 | 0.037 | 0.071 | <0.001 |
| Avoidance of private appointments | Italian | July 6, 2020 to October 18, 2020 | 0.006 | 0.004 | 0.009 | <0.001 |
| Avoidance of private appointments | Italian | October 19, 2020 to January 17, 2021 | 0.053 | 0.038 | 0.074 | <0.001 |
| Avoidance of private appointments | Italian | January 18, 2021 onwards | 0.026 | 0.018 | 0.037 | <0.001 |
| Non-use of public transport | German/Romansh | March 16, 2020 to May 10, 2020 | 1.000 | NA | NA | NA |
| Non-use of public transport | German/Romansh | May 11, 2020 to July 5, 2020 | 0.231 | 0.231 | 0.231 | <0.001 |
| Non-use of public transport | German/Romansh | July 6, 2020 to October 18, 2020 | 0.052 | 0.052 | 0.052 | <0.001 |
| Non-use of public transport | German/Romansh | October 19, 2020 to January 17, 2021 | 0.144 | 0.144 | 0.144 | <0.001 |
| Non-use of public transport | German/Romansh | January 18, 2021 onwards | 0.124 | 0.124 | 0.124 | <0.001 |
| Non-use of public transport | French | March 16, 2020 to May 10, 2020 | 1.000 | NA | NA | NA |
| Non-use of public transport | French | May 11, 2020 to July 5, 2020 | 0.159 | 0.116 | 0.217 | <0.001 |
| Non-use of public transport | French | July 6, 2020 to October 18, 2020 | 0.027 | 0.019 | 0.038 | <0.001 |
| Non-use of public transport | French | October 19, 2020 to January 17, 2021 | 0.073 | 0.052 | 0.101 | <0.001 |
| Non-use of public transport | French | January 18, 2021 onwards | 0.042 | 0.030 | 0.060 | <0.001 |
| Non-use of public transport | Italian | March 16, 2020 to May 10, 2020 | 1.000 | NA | NA | NA |
| Non-use of public transport | Italian | May 11, 2020 to July 5, 2020 | 0.119 | 0.077 | 0.184 | <0.001 |
| Non-use of public transport | Italian | July 6, 2020 to October 18, 2020 | 0.024 | 0.015 | 0.039 | <0.001 |
| Non-use of public transport | Italian | October 19, 2020 to January 17, 2021 | 0.061 | 0.039 | 0.096 | <0.001 |
| Non-use of public transport | Italian | January 18, 2021 onwards | 0.043 | 0.027 | 0.068 | <0.001 |
