# Supplementary material for: Health and social behaviour through pandemic phases in Switzerland: Regional time-trends of the COVID-19 Social Monitor panel study
Source: PLoS One. 2021 Aug 25;16(8):e0256253. doi: 10.1371/journal.pone.0256253 (PMC8386858; doi:10.1371/journal.pone.0256253)
Supplement: S1 Text — (PDF) [file pone.0256253.s007.pdf]

S2 Text. Statistical methods for calibration weights.

We describe the survey population and calibrations weights by frequencies (n), percentages (%) and box plots. We calculate 95% confidence intervals (CI) for proportions based on the method from Clopper and Pearson. Let $\mathcal{O}_{1355} = (\mathcal{O}_{1,1355}, ..., \mathcal{O}_{1355,1355})^T$ be the set of observed outcomes from the additional December 2020 survey sample of 1,355 participants and $\mathcal{O}_{2026} = (\mathcal{O}_{1,2026}, ..., \mathcal{O}_{2026,2026})^T$ be the set of observed outcomes from the March 2020 survey sample. Because individuals were randomly selected we assume that complete set of observed variables $\mathcal{O}_{3381} = \{\mathcal{O}_{1355}, \mathcal{O}_{2026}\}$ are realisation from identically and independently distributed random variables. We denote the combined initial March 2020 survey sample of 2,026 participants and the additional 1,355 participants from the December 2020 survey sample as December* 2020 sample. Note that - because of nonresponse of participants from the March 2020 survey - the December* 2020 survey sample is actually not fully observed.

Let $Y_{il,u}$ denote the binary outcome whether individual $i \leq n_u$ living in language region $l = \{1, 2, 3\}$ was sampled from the Swiss population aged 15 years or older in 2018 in survey wave $u \in \{1 = March,\ 2 = December\}$. We construct sampling weights $SW_{i,u} := SW_{il,u} = 1/Prob(Y_{il,u} = 1)$ for $i \leq n_u$, $l = \{1, 2, 3\}$, $u \in \{1, 2\}$. We specify a hierarchical logistic regression model (M1) as

$$(\mathbf{M1}) \quad Y_{il,u} = \beta_0 + \beta^T agecat * gender + lregion_l + \epsilon_{il}, \quad i \leq n_u,\ l = \{1, 2, 3\},\ u \in \{1, 2\},$$

where n_u is the survey sample size in u, β_0 is the overall intercept, $\beta^T = (\beta_1, \beta_2, ..., \beta_5)$ is a vector of estimates from an interaction term (a+b+a:b) between age category (*agecat1*: <45 years, *agecat2*: 45-<65 years, *agecat3*: 65+ years) and gender (*female*: 0 Men, 1 Women), and $lregion_l \sim N(0, \tau_{lregion})$ is an unstructured random effect for the variable language region (*lregion*: 1 German/Romansh, 2 French, 3 Italian). We use a Bayesian modeling approach using noninformative centered Gaussian priors for β_0, β^T, i.e. $p(\beta_z|\mu_z, \sigma_z^2) = N(0, 1000)$, $z \leq 5$, and a centered Gaussian distributed prior with an inverse-Gamma distributed log precision parameter $\tau_{lregion} \sim \Gamma^{-1}(1, 5/100000)$ for the unstructured random effect.

As sensitivity analyses we perform 1) a Bayesian hierarchical logistic model (M2) with age category, gender and language region as random effects, 2) a Bayesian non-hierarchical logistic regression model (M3) with age category, gender and language region as fixed effects, 3) a frequentist logistic regression model (see the appendix section sensitivity analysis in this document). We compare performances of models (M1), (M2) and (M3) by Watanabe–Akaike information criterion (WAIC) with a lower WAIC indicating better model performance.

Let $Z_{iw,u} \in \{0, 1\}$ denote the binary outcome whether individual $i \leq n_u$ participated in survey wave $w \geq 2$, $u \in \{1, 2\}$. We define a Bayesian logistic regression model for the construction of nonresponse weights as follows

$$Z_{iw,u} = \gamma_0 + \gamma_1 agecat + \gamma_2 gender + \gamma_3 lregion + \gamma_4 partner + \gamma_5 work + \gamma_6 education + \epsilon_{iw}, \quad i \leq n_u,\ w \geq 2,\ u \in \{1, 2\},$$

where n_u is the survey sample size in u, with additional variables living with partner (*partner*: 0 No, 1 Yes), working situation (*work*: 1 Employed, 2 Unemployed, 3 Retired, 4 Other (e.g. apprenticeship) and highest attained education (*educ*: 1 Compulsory, 2 Secondary, 3 Tertiary). We use noninformative centered Gaussian priors for γ_z, $z \leq 5$, i.e. $p(\gamma_z|\theta_i, \rho_i^2) = N(0, 1000)$, $z \leq 5$. We construct nonresponse weights as $NRW_{iw,u} = 1/(1 - Prob(Z_{iw,u} = 1))$ for $i \leq n_u$, $u \in \{1, 2\}$, $w \geq 2$. Calibration weights are defined as $CW_{iw,u} = NRW_{iw,u} \cdot SW_{i,u}$, $i \leq n_u$, $u \in \{1, 2\}$, $w \geq 2$. For Bayesian calculations and model building we used the Integrated Nested Laplace Approximation (INLA) approach (R INLA (https://www.r-inla.org/)).

Construction of sampling weights for survey waves in December 2020 and onwards

agecat	female	lregion	pred_ci

<45	Women	German/Romansh	0.046%, 95% CI (0.043%, 0.05%)
45- <65	Women	German/Romansh	0.044%, 95% CI (0.041%, 0.048%)
65+	Women	German/Romansh	0.026%, 95% CI (0.023%, 0.03%)
<45	Men	German/Romansh	0.049%, 95% CI (0.046%, 0.053%)
45- <65	Men	German/Romansh	0.045%, 95% CI (0.041%, 0.049%)
65+	Men	German/Romansh	0.033%, 95% CI (0.029%, 0.037%)
<45	Women	French	0.045%, 95% CI (0.041%, 0.049%)
45- <65	Women	French	0.043%, 95% CI (0.039%, 0.047%)
65+	Women	French	0.025%, 95% CI (0.022%, 0.029%)
<45	Men	French	0.048%, 95% CI (0.043%, 0.052%)
45- <65	Men	French	0.043%, 95% CI (0.039%, 0.048%)
65+	Men	French	0.032%, 95% CI (0.028%, 0.037%)
<45	Women	Italian	0.177%, 95% CI (0.158%, 0.196%)
45- <65	Women	Italian	0.168%, 95% CI (0.15%, 0.188%)
65+	Women	Italian	0.1%, 95% CI (0.085%, 0.115%)
<45	Men	Italian	0.187%, 95% CI (0.168%, 0.208%)
45- <65	Men	Italian	0.17%, 95% CI (0.151%, 0.19%)
65+	Men	Italian	0.126%, 95% CI (0.109%, 0.145%)

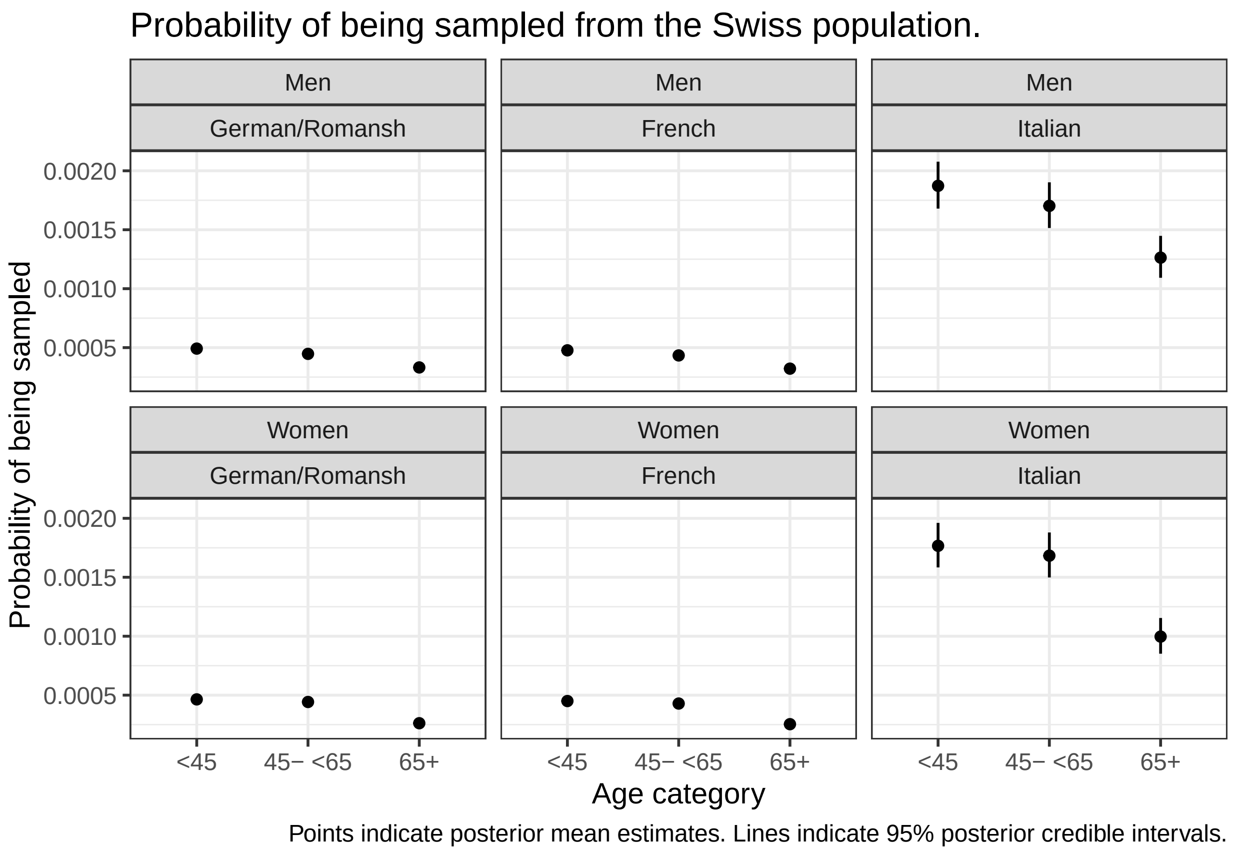

A box plot of the sampling weights for the December* 2020 survey sample and the recalibration sampling weights for the March 2020 survey sample are shown in the next Figure. For the December 2020 survey sample the median of sampling weights is 2155, the 1st quantile 2033, the third quantile 2261, minimum 534, maximum 3939. For the March 2020 survey sample the median of the sampling weights is 3614, the 1st quantile 3359, the third quantile 3799, minimum 900, maximum 6396.

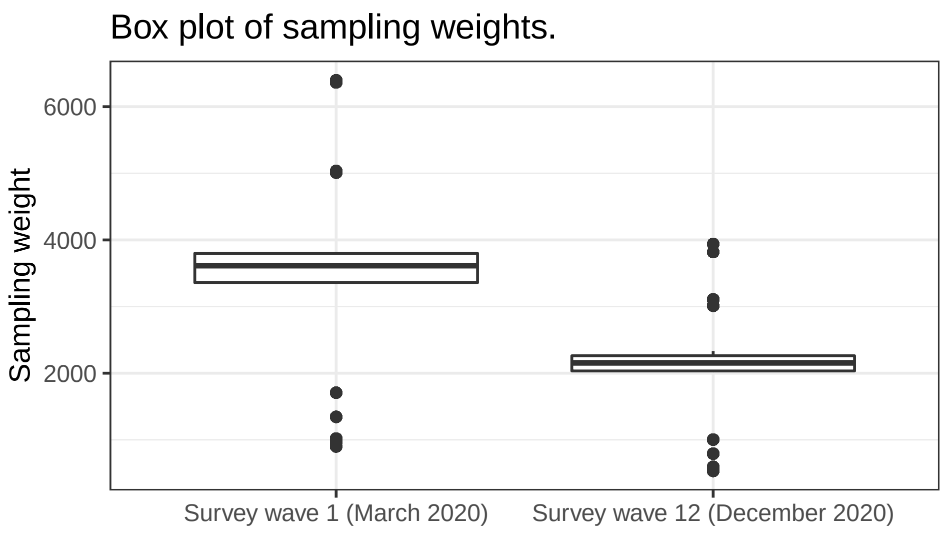

We used the sampling design based on age categories, gender and language region to reconstruct the Swiss population based on the sampling weights. The following table shows the sampling weighted estimated counts with 95% CI (column *Weighted estimate*, *lci*, *uci*).

agecat	female	lregion	Weighted estimate	lci	uci	num_pop
<45	Men	German/Romansh	1120183	1043386	1196980	1138490
45- <65	Men	German/Romansh	856388	785786	926990	851030
65+	Men	German/Romansh	551196	487328	615064	535498
<45	Women	German/Romansh	1116290	1036223	1196357	1141840
45- <65	Women	German/Romansh	863702	792359	935045	853534
65+	Women	German/Romansh	557574	481212	633936	537073
<45	Men	French	387760	335017	440503	399229
45- <65	Men	French	269802	223369	316235	275497
65+	Men	French	177099	133747	220451	167685
<45	Women	French	404404	348891	459917	411995
45- <65	Women	French	284504	236667	332341	284307
65+	Women	French	196950	145075	248825	173047
<45	Men	Italian	67818	56472	79164	60468
45- <65	Men	Italian	54004	43401	64607	54279
65+	Men	Italian	32472	22934	42010	39374
<45	Women	Italian	71316	59334	83298	62992
45- <65	Women	Italian	53460	42838	64082	56544
65+	Women	Italian	29087	18798	39376	41017

Calibration weights

The next figure shows the box plots of calibration weights, by survey wave.

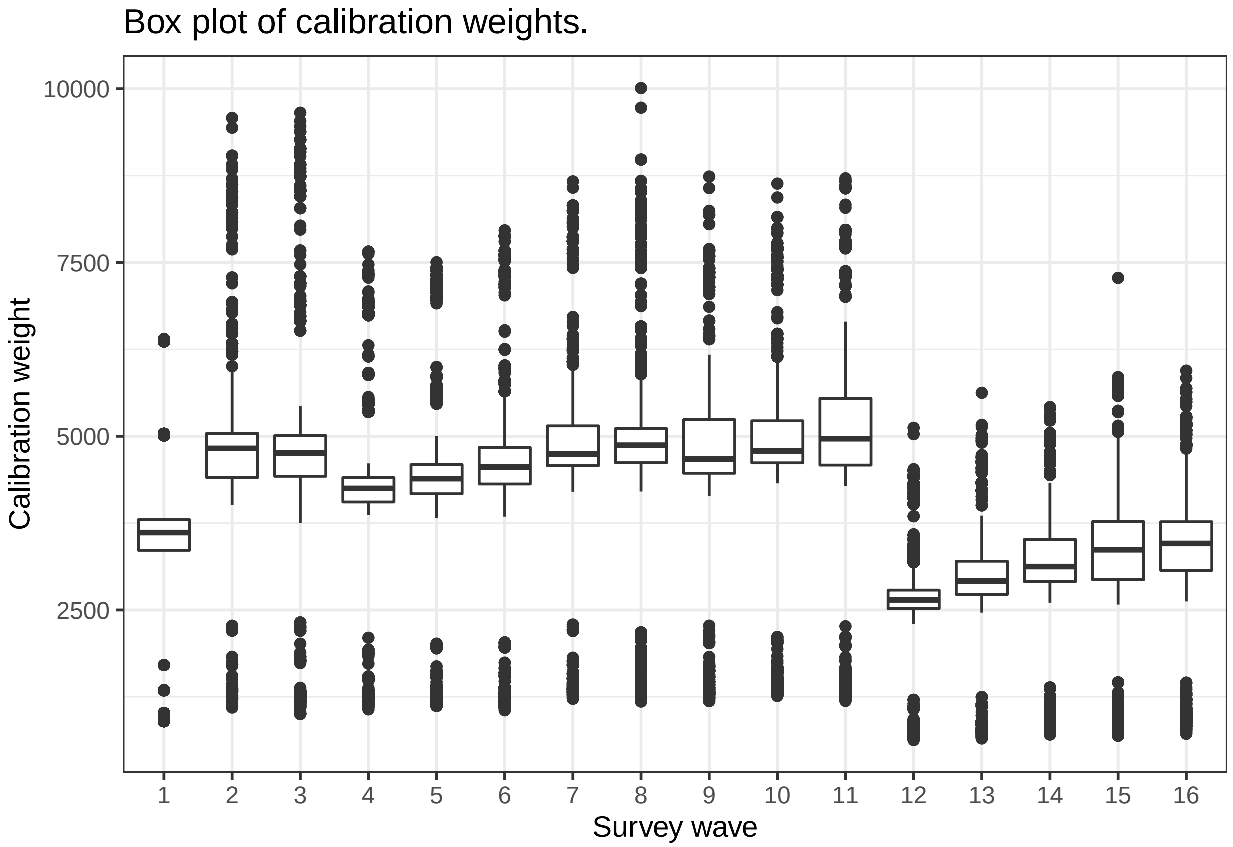

The next table describes the sum of calibration weights for each survey wave. This sum should approximate the underlying 2018 census population of Switzerland aged 15 years or older (N=7.1 million individuals).

wave	calib_weight
1	7088561
2	7100180
3	7093499
4	7097338
5	7099392
6	7096689
7	7086740
8	7095359
9	7090656
10	7095338
11	7091904
12	7098594
13	7097538
14	7095351
15	7088897
16	7094197

Sensitivity analyses

We perform three sensitivity analyses:

- 1) A model approach with age category, gender and language region as random effects,
- 2) A model approach with age category, gender and language region as fixed effects,
- 3) We compare estimates from the Bayesian approach with a frequentist approach.

We compare WAIC of sensitivity analyses 1) and 2), and the model in the main section. Without loss of generality we use only the December 2020 survey sample to assess model performance.

Sensitivity analysis 1)

Let Y_{iasl} denote the binary outcome whether individual $i \leq 3381$ with age in age category $a = \{1, 2, 3\}$, gender $s = \{1, 2\}$ and living in language region $l = \{1, 2, 3\}$ was sampled from the Swiss population aged 15 years or older in 2018. We specify a Bayesian hierarchical logistic regression model (M2) as

$$(\mathbf{M2}) \quad Y_{iasl} = \beta_0 + agecat_a + gender_s + lregion_l + \epsilon_{iasl}, \quad i \leq 3381,\ a = \{1, 2, 3\},\ s = \{1, 2\},\ l = \{1, 2, 3\},$$

where β_0 is the overall intercept, and $agecat_a \sim N(0, \tau_{agecat})$, $gender_s \sim N(0, \tau_{gender})$, $lregion_l \sim N(0, \tau_{lregion})$ are unstructured random effects for variables age category (*agecat1*: <45 years, *agecat2*: 45- <65 years, *agecat3*: 65+ years), gender (*female*: 0 Men, 1 Women) and language region (*lregion*: 1 German/Romansh, 2 French, 3 Italian). We use a noninformative centered Gaussian prior for β_0, i.e. $p(\beta_0|\mu_0, \sigma_0^2) = N(0, 1000)$ and that the prior for the unstructured random effects are centered Gaussian distributed with inverse-Gamma distributed log precision parameters $\tau_{agecat}, \tau_{gender}, \tau_{lregion} \sim \Gamma^{-1}(1, 5/100000)$.

Sensitivity analysis 2)

Let $Y_i \in \{0, 1\}$ denote the binary outcome whether individual $i \leq 3381$ is sampled from the Swiss population aged 15 years or older in 2018. We define a non-hierarchical logistic regression model (M3) as

$$(\mathbf{M3}) \quad Y_i = \beta_0 + \beta^T agecat * female * lregion + \epsilon_i, \quad i \leq 3381,$$

where β_0 is the overall intercept, and $\beta^T = (\beta_1, \beta_2, ..., \beta_{17})$ is a vector of estimates from an interaction term (a+b+c+a:b+a:c+b:c) between variables age category (*agecat1*: <45 years, *agecat2*: 45- <65 years, *agecat3*: 65+ years), gender (*female*: 0 Men, 1 Women) and language region (*lregion*: 1 German/Romansh, 2 French, 3 Italian). We use a Bayesian approach with noninformative centered Gaussian priors for β_z, $z \leq 17$, i.e. $p(\beta_z|\mu_z, \sigma_z^2) = N(0, 1000)$, $z \leq 17$.

The following table shows the model performance measured by WAIC for the specified models (M1), (M2) and (M3).

model	WAIC
Main model (M1)	147.9
Sensitivity model (M2)	152.5
Sensitivity model (M3)	151.9

The specified model in the main analysis (M1) showed the lowest WAIC, i.e. best model performance (WAIC=147.9).

Sensitivity analysis 3)

The following table shows estimates and 95% CI from a frequentist logistic regression model (columns *est_glm*, *lci_glm*, *uci_glm*) and posterior means and 95% credible intervals from the Bayesian model of the specified model in (M3) (columns *est_inla*, *lci_inla*, *uci_inla*).

	est_glm	lci_glm	uci_glm	est_inla	lci_inla	uci_inla
(Intercept)	-7.633	-7.717	-7.550	-7.633	-7.718	-7.550
factor(agecat)2	-0.073	-0.203	0.058	-0.073	-0.204	0.058
factor(agecat)3	-0.348	-0.515	-0.181	-0.348	-0.518	-0.182
female	-0.065	-0.185	0.055	-0.065	-0.185	0.056
factor(lregion)2	-0.043	-0.210	0.123	-0.043	-0.213	0.122
factor(lregion)3	1.468	1.275	1.661	1.469	1.272	1.660
factor(agecat)2:female	0.059	-0.127	0.245	0.059	-0.127	0.245
factor(agecat)3:female	-0.164	-0.413	0.084	-0.164	-0.414	0.084
factor(agecat)2:factor(lregion)2	-0.015	-0.280	0.251	-0.014	-0.282	0.251
factor(agecat)3:factor(lregion)2	0.038	-0.303	0.379	0.038	-0.308	0.375
factor(agecat)2:factor(lregion)3	-0.142	-0.440	0.157	-0.142	-0.443	0.156
factor(agecat)3:factor(lregion)3	-0.354	-0.744	0.036	-0.355	-0.753	0.029
female:factor(lregion)2	0.017	-0.220	0.254	0.017	-0.221	0.255
female:factor(lregion)3	0.016	-0.258	0.290	0.016	-0.259	0.291
factor(agecat)2:female:factor(lregion)2	-0.001	-0.376	0.374	-0.001	-0.377	0.375
factor(agecat)3:female:factor(lregion)2	0.049	-0.448	0.547	0.050	-0.450	0.548
factor(agecat)2:female:factor(lregion)3	-0.073	-0.497	0.351	-0.073	-0.498	0.352
factor(agecat)3:female:factor(lregion)3	-0.174	-0.765	0.416	-0.175	-0.771	0.413

The frequentist model reveals similar estimates as the Bayesian approach in the main analysis.
