# Supplementary material for: Health and social behaviour through pandemic phases in Switzerland: Regional time-trends of the COVID-19 Social Monitor panel study
Source: PLoS One. 2021 Aug 25;16(8):e0256253. doi: 10.1371/journal.pone.0256253 (PMC8386858; doi:10.1371/journal.pone.0256253)
Supplement: S2 Text — (PDF) [file pone.0256253.s008.pdf]

S8 Text. Reproducible analysis example.

This document describes the main analysis steps in the manuscript, restricted to the variable hb8_3_cat1 (wearing of face mask), as example. For privacy preserving reasons we do not provide individual patient identifiers, no individual wave identifier, age in categories and no working employment information. Missing values were replaced (as described in the methods section) by its median values. Thus, no code example from an hierarchical model (as used in the main manuscript) can be provided. We used instead a oridnary survey weighted regression.

Used statistical software: R

```
library(tidyverse)
library(survey)
library(reporttools)
library(lme4)

# Read data
data <- read_csv(paste0(main.path, "Data.csv"))

# Descriptive table
data_report <- data.frame(data %>% filter(!is.na(wave_id))
                          %>% select(agecat, female, lregion, education, nat, partner, urban))

tableNominal(data_report[, -which(names(data_report)=="lregion")]
             , group=data_report$lregion, cumsum = F,
             longtable = F, cap="Characteristics of study population")
```

Variable	Levels	n_1	$\%_1$	n_2	$\%_2$	n_3	$\%_3$	n_{all}	$\%_{all}$
agecat	1	1069	49.4	367	51.5	253	50.1	1689	50.0
	2	765	35.4	239	33.5	182	36.0	1186	35.1
	3	329	15.2	107	15.0	70	13.9	506	15.0
	all	2163	100.0	713	100.0	505	100.0	3381	100.0
female	0	1117	51.6	359	50.4	260	51.5	1736	51.4
	1	1046	48.4	354	49.6	245	48.5	1645	48.6
	all	2163	100.0	713	100.0	505	100.0	3381	100.0
education	1	154	7.1	55	7.7	23	4.5	232	6.9
	2	1038	48.0	342	48.0	258	51.1	1638	48.5
	3	971	44.9	316	44.3	224	44.4	1511	44.7
	all	2163	100.0	713	100.0	505	100.0	3381	100.0
nat	0	150	6.9	91	12.8	55	10.9	296	8.8
	1	2013	93.1	622	87.2	450	89.1	3085	91.2
	all	2163	100.0	713	100.0	505	100.0	3381	100.0
partner	0	636	29.4	235	33.0	127	25.1	998	29.5
	1	1527	70.6	478	67.0	378	74.8	2383	70.5
	all	2163	100.0	713	100.0	505	100.0	3381	100.0
urban	0	484	22.4	123	17.2	50	9.9	657	19.4
	1	1679	77.6	590	82.8	455	90.1	2724	80.6
	all	2163	100.0	713	100.0	505	100.0	3381	100.0

Table 1: Characteristics of study population

```
# Set survey design and calibration weights
# Note: No personal identifier can be deliviered because of privacy protection reasons
# Thus: No clustering assumed id=~1
wdesign <- svydesign(id=~1, strata=~agecat+female+lregion, weights=~calib_weight, data=data)

# Proportion in wave_cat 1 and lregion 1
mod_0 <- svyglm(hb8_3_cat1~1, data=data, family=binomial(), design=wdesign,
                subset=wave_cat==1 & lregion==1)
```

```r
plogis(mod_0$coefficients)
```

```
## (Intercept)
##  0.01251584
```

```r
# Proportion in wave_cat 4 and lregion 1
mod_0 <- svyglm(hb8_3_cat1~1, data=data, family=binomial(), design=wdesign,
                subset=wave_cat==4 & lregion==1)
plogis(mod_0$coefficients)
```

```
## (Intercept)
##     0.77109
```

```r
# Test for interaction effect
mod_0 <- svyglm(hb8_3_cat1~factor(wave_cat)+factor(lregion), data=data,
                family=binomial(), design=wdesign)
mod_1 <- svyglm(hb8_3_cat1~factor(wave_cat)*factor(lregion), data=data,
                family=binomial(), design=wdesign)
round(anova(mod_0, mod_1)$p,3)
```

```
## [1] 0
```

```r
# Test for time effect in stratified model (here lregion==1)
mod_0 <- svyglm(hb8_3_cat1~1, data=data, family=binomial(),
                design=wdesign, subset=lregion==1)
mod_1 <- svyglm(hb8_3_cat1~factor(wave_cat), data=data, family=binomial(),
                design=wdesign, subset=lregion==1)
round(anova(mod_0, mod_1)$p,3)
```

```
## [1] 0
```

```r
# Test for time effect in stratified model (here lregion==1), adjusted
mod_0 <- svyglm(hb8_3_cat1~factor(agecat)+factor(female)+factor(education)+
                factor(urban)+factor(nat)+factor(partner), data=data,
                family=binomial(), design=wdesign, subset=lregion==1)
mod_1 <- svyglm(hb8_3_cat1~factor(wave_cat)+factor(agecat)+factor(female)+
                  factor(education)+factor(urban)+factor(nat)+factor(partner),
                data=data, family=binomial(), design=wdesign, subset=lregion==1)
round(anova(mod_0, mod_1)$p,3)
```

```
## [1] 0
```
