# Supplementary material for: Health and social behaviour through pandemic phases in Switzerland: Regional time-trends of the COVID-19 Social Monitor panel study
Source: PLoS One. 2021 Aug 25;16(8):e0256253. doi: 10.1371/journal.pone.0256253 (PMC8386858; doi:10.1371/journal.pone.0256253)
Supplement: S3 Text — (PDF) [file pone.0256253.s009.pdf]

S9 Text. Codebook.

Variable name	Variable levels	Variable labels	Remark
agecat	1	0-45 years	
	2	45-65 years	
	3	65+ years	
female	0	Men	
	1	Women	
lregion	1	German/Romansh	
	2	French	
	3	Italian	
education	1	Compulsory	
	2	Secondary	
	3	Tertiary	
nat	1	Swiss	
	0	Non-Swiss	
partner	1	Living with partner	
	0	Not living with partner	
urban	1	Living in urban area	
	0	Living in rural area	
wave_cat	1	Mitigation period to May 10, 2020	
	2	Mitigation period May 11, 2020 to July 5, 2020	
	3	Mitigation period July 6, 2020 to October 18, 2020	
	4	Mitigation period October 19, 2020 onwards	
wave_id	1	Wave 1 participants	
	12	Additional wave 12 participants	
ph1_cat2	1	Poor to very poor health status	
wb2_cat2	1	Poor to very poor quality of life	
ms2_2_4_cat1	1	Presence of depressive mood	
ms2_6_cat1	1	Lack of energy	
wc1_cat	1	Fears of loosing employement	
ss3_cat1	1	Feelings of loneliness	
eh_3_3_cat1	1	Feelings of social isolation (65 years or older)	Only for 65 years or older; not asked in first survey wave
hb7_cat	1	No physical activity	
hs1_1_cat	1	Health care use	
hs2_1_cat	1	Health care non-use	
hs4_cat	1	COVID-19 related health care use	
hb8_2_cat1	1	Physical distance	Not asked in first survey wave
hb8_3_cat1	1	Wearing of face mask	Not asked in first survey wave
hb8_4_cat1	1	Avoidance of private appointments	Not asked in first survey wave
hb8_5_cat1	1	Non-use of public transport	Not asked in first survey wave
calib_weight	>0	Calibration weight	
