# Supplementary material for: Health and social behaviour through pandemic phases in Switzerland: Regional time-trends of the COVID-19 Social Monitor panel study
Source: PLoS One. 2021 Aug 25;16(8):e0256253. doi: 10.1371/journal.pone.0256253 (PMC8386858; doi:10.1371/journal.pone.0256253)
Supplement: Attachment — Submitted filename: Letter_R2.docx [file pone.0256253.s011.docx]

Re: “Health and social behaviour through pandemic phases in Switzerland: Longitudinal analysis of the COVID-19 Social Monitor panel study”

Winterthur, Juli 22 2021

Dear Prof. Dr. Guidi

We thank you for the opportunity to submit a revised version of our manuscript to PLOS One. Please find below our point-by-point answers to the comments of reviewer #1. Reviewers #2 and #3 had no further comments which need to be addressed in the revised manuscript.

Based on the comment of reviewer #1 we changed the statistical analysis approach towards a hierarchical regression model which accounts for repeated measurements within individuals. Our previous analysis strategy used a cluster-robust approach for addressing repeated measurements which was criticized by reviewer #1. Compared to our previous analyses we found evidence for an interaction effect for the study outcomes poor quality of life (p<0.001), depressive mood (p=0.005), no physical activity (p=0.04), besides the already reported adherence to mitigation measures (p<0.001), from adjusted hierarchical logistic regression models. These findings do not change our previous discussion or interpretation and can likely be explained by the correlation structure between repeated measurements.

Further reviewer #1 required to add p-values for analyses. We added p-values from adjusted regression models (S7 Table).

We updated our discussion about socioeconomic factors with a recently published study which investigated the inverse care law in Switzerland (doi.org/10.1016/S2468-2667(21)00160-2).

We hope that the Editor and reviewer #1 are satisfied with our newly revised manuscript.

**Yours sincerely**

**Marc Höglinger, PhD (on behalf of all co-authors)**

PONE-D-21-02911R1

Health and social behaviour through pandemic phases in Switzerland: Regional time-trends of the COVID-19 Social Monitor panel study

PLOS ONE

**[ No additional revision points has been raised by the Editor. ]**

6. Review Comments to the Author

Reviewer #1: The paper has two main problems that the authors did not address:

First, the statistical method applied inappropriately; second, the statistical interpretation of the results is incorrect.

**We appreciate the thoughtful feedback and time efforts of reviewer #1.**

1- For investigating the pattern of change during the time, the statistical model must have the ability to investigate the change of outcome during the time, but the logistic regression that was applied in this study is only able to estimate the outcome (odds ratio in this paper) for the specific time point and cannot compare the odds ratios in different time points to show whether the pattern of change exists or not; for showing the pattern of change during the time the longitudinal study should be applied.

**Our previous model approach is mentioned in classical statistical textbooks for longitudinal analyses like, for instance, Fixed Effects Regression Methods for Longitudinal Data Using SAS from Paul Allison (ISBN 978-1-59047-568-3), Chapter 3.3. Estimation of Logistic Models for Two or More Observations Per Person. In our previous analysis strategy, we accounted for repeated measurements on individuals with a cluster-robust approach.**

**Based on the comments from reviewer #1, we changed our modeling strategy to a hierarchical logistic regression model which accounts for repeated measurements on the same individual. That is, we account for nested repeated measurements within individuals while investigating a common fixed effect over periods (Reference: Models for Discrete Longitudinal Data from Molenberghs and Verbeke, doi: 10.1007/0-387-28980-1).**

**We note that an odds ratio inherently has a reference group, i.e., one compares the odds of the presence of the study outcome of a specific variable category (say, time period 3) with the odds of the reference category (say, time period 1). In our case the reference period is the first period as defined in the manuscript. Thus, our odds ratio estimates are not only for one specific time point – as reviewer #1 says - but compares subsequent time periods with the reference period.**

2- Frequentist statistics uses P-value as a measure of probability to show that the effect exists and the observed effect is statistically meaningful; judging about the existence of effect is not subjective and cannot be shown without P-value (or confidence interval), so reporting p-value is not an optional choice.

Of course, there are some drawback to frequentist inference including the p-value approach to hypothesis testing, but it’s the only method for frequentists to test their hypothesis; The ASA statement is informing researchers to be aware of problems related to p-value and warn them to be cautious about the interpretation of the results; but without having the alternative to p-value, it cannot be omitted from the results; if a researcher does not accept the frequentist approach and their use of p-value for hypothesis testing, he/she can apply another branch of statistics, called Bayesian inference, that addresses some limitation of frequents approach including the use of p-value; otherwise, if the frequentist method is used to analyze the data, their rules must be followed exactly.

**We added to the statistical section that we defined a 5% alpha level as statistically significant and reported p-values in S7 Table. All other p-values for interactions and time effects have already been reported.**

The Authors referred to a checklist they used to organize their results in order to justify their reported outcome, however, the checklist only mentioned the key points that must be reported in observational studies and does not explain the details of the application of the statistical method and interpretation of the results; for applying the statistical method correctly and vigorously, the authors need to refer to statistical textbooks.

**As mentioned in the previous rebuttal letter, we based our reasoning to focus on effect size estimation (rather than p-value based assessments of significance) on different statistical textbooks (Essential Medical Statistics by Kirkwood and Sterne, ISBN 978-0-865-42871-3), “Likelihood and Bayesian Inference” from Held and Sabanés Bové (ISBN 978-3-662-60792-3), and “Modern Epidemiology” form Lash, VanderWeele, Haneuse, Rothman (ISBN 978-3-662-60792-3).**

Reviewer #2: Thank you for the good work in addressing my comments which were addressed to my satisfaction. I believe this manuscript will make an important contribution to the ongoing and rapidly developed COVID19 literature.

**We thank reviewer #2 for the helpful comments and feedback, and highly appreciate the time efforts for the review. We note that we changed the statistical analysis approach towards a hierarchical regression model which accounts for repeated measurements within individuals. Our previous analysis strategy used a cluster-robust approach for addressing repeated measurements. Further, we updated our discussion about socioeconomic factors with a recently published study which investigated the inverse care law in Switzerland (doi.org/10.1016/S2468-2667(21)00160-2).**

Reviewer #3: This manuscript adds to the literature health and social behavior changes associated with COVID-19 and provides a unique angle by examining differences in adherence by language regions. The authors addressed reviewer comments appropriately in the manuscript. All data are fully available online without restriction.

**Thank you. We believe that your feedback substantially improved our manuscript, and we highly appreciate the time efforts for the review. We note that we changed the statistical analysis approach towards a hierarchical regression model which accounts for repeated measurements within individuals. Our previous analysis strategy used a cluster-robust approach for addressing repeated measurements. Further, we updated our discussion about socioeconomic factors with a recently published study which investigated the inverse care law in Switzerland (doi.org/10.1016/S2468-2667(21)00160-2).**

**We thank the Editor and the reviewers for their helpful feedback which substantially improved our manuscript.**

**Marc Höglinger (and Co-authors)**
